# Breeding Value of Primary Synthetic Wheat Genotypes for Grain Yield

**DOI:** 10.1371/journal.pone.0162860

**Published:** 2016-09-22

**Authors:** Jafar Jafarzadeh, David Bonnett, Jean-Luc Jannink, Deniz Akdemir, Susanne Dreisigacker, Mark E. Sorrells

**Affiliations:** 1 Department of Plant Breeding and Genetics, Cornell University, Ithaca, NY, United States of America; 2 International Maize and Wheat Improvement Center (CIMMYT), Apdo. Postal 6-641, 06600 El Batan, Mexico; 3 USDA-ARS, R.W. Holley Center for Agriculture and Health, Ithaca, NY, United States of America; National Institute for Plant Genome Research, INDIA

## Abstract

To introduce new genetic diversity into the bread wheat gene pool from its progenitor, *Aegilops tauschii* (Coss.) Schmalh, 33 primary synthetic hexaploid wheat genotypes (SYN) were crossed to 20 spring bread wheat (BW) cultivars at the International Wheat and Maize Improvement Center. Modified single seed descent was used to develop 97 populations with 50 individuals per population using first back-cross, biparental, and three-way crosses. Individuals from each cross were selected for short stature, early heading, flowering and maturity, minimal lodging, and free threshing. Yield trials were conducted under irrigated, drought, and heat-stress conditions from 2011 to 2014 in Ciudad Obregon, Mexico. Genomic estimated breeding values (GEBVs) of parents and synthetic derived lines (SDLs) were estimated using a genomic best linear unbiased prediction (GBLUP) model with markers in each trial. In each environment, there were SDLs that had higher GEBVs than their recurrent BW parent for yield. The GEBVs of BW parents for yield ranged from -0.32 in heat to 1.40 in irrigated trials. The range of the SYN parent GEBVs for yield was from -2.69 in the irrigated to 0.26 in the heat trials and were mostly negative across environments. The contribution of the SYN parents to improved grain yield of the SDLs was highest under heat stress, with an average GEBV for the top 10% of the SDLs of 0.55 while the weighted average GEBV of their corresponding recurrent BW parents was 0.26. Using the pedigree-based model, the accuracy of genomic prediction for yield was 0.42, 0.43, and 0.49 in the drought, heat and irrigated trials, respectively, while for the marker-based model these values were 0.43, 0.44, and 0.55. The SYN parents introduced novel diversity into the wheat gene pool. Higher GEBVs of progenies were due to introgression and retention of some positive alleles from SYN parents.

## Introduction

Domestication and breeding of wheat for many years has increased yield, but recently this increase has slowed down, in part, due to the reduction of genetic variation in the cultivated wheat gene pool [1]. Bread wheat (*Triticum aestivum* L.) originated by natural hybridization between durum wheat (*Triticum*. *turgidum* L. subsp. *durum*) and *Aegilops tauschii* (Coss.) Schmalh, but this probably only happened one or a few times and involved only a few progenitors. Consequently, potential genetic diversity in durum and *Ae*. *tauschii* was not represented in bread wheat germplasm [1,2]. One approach to introducing new genetic diversity into the cultivated bread wheat gene pool from wheat progenitors is to develop and use synthetic hexaploid wheat (SYN) in breeding [3]. The SYNs are amphiploids resulting from interspecific crosses between a diploid *Ae*. *tauschii*, donor of the D genome and a modern durum or emmer wheat (*Triticum turgidum* L. subsp. *dicoccum)* wheat donor of the A and B genomes. About 1200 winter and spring habit SYN lines have been developed at the International Maize and Wheat Improvement Center (CIMMYT) since the 1980s [4]. Using SYNs, considerable genetic diversity has been captured from the progenitors of bread wheat [3,5]. The practical value of this diversity can be seen in the resistance to a range of biotic stresses such as Karnal bunt (*Tilletia indica* Mitra) [6], stripe rust (*Puccinia striiformis* f. sp. xtritici) [7], Septoria tritici blotch (*Mycosphaerella graminicola* (Fückel.) J. Schröt in Cohn) [8], cereal cyst nematode (*Heterodera avenae* Wollenweber) [9] and stem rust (*Puccinia graminis* Pers.:Pers. f. sp. *tritici* Eriks. E. Henn.) [10]. Also, SYNs are a valuable genetic resource for abiotic stress such as drought [11]. Lopes and Reynolds [11] reported that synthetic derived wheat lines (SDLs) increased drought tolerance which was attributed to traits such as earlier flowering, greater root mass at depth, greater water extraction capacity, and increased water use efficiency at anthesis to produce an average of 26% higher grain yield than the cultivated wheat parents under terminal drought. Hence, crossing SYNs to modern wheat cultivars could result in more productive cultivars for such stress environments. Furthermore, studying yield potential of synthetic backcross-derived lines (SBLs) in the diverse rain-fed environments of Australia showed that SBLs out-yielded the best local checks by 8 to 30% [12]. Cooper et al.[13] backcrossed ten elite primary synthetics to two Texas winter wheat cultivars, TAM111 and TAM112, and evaluated SBLs for yield and yield components. They reported that improved yield in the SBLs was due to an increased number of heads per unit area and grains per head.

In China, SYN lines have been used in breeding programs and four synthetic derived cultivars, Chuanmai 38, Chuanmai 42, Chuanmai 43 and Chuanmai 47 were released and are widely grown by farmers. Of these, Chuanmai 42 had large kernels, resistance to stripe rust, and its grain yield was 16.4 to 22.7% higher than the commercial check, Chuanmai 107 [14,15].

Molecular markers can be used to evaluate the diversity within and among germplasms and to monitor genetic diversity over time [16–18]. Also, molecular markers allow more accurate prediction of breeding values of genotypes through improved estimates of relatedness and estimation of marker effects[19]. These values can be used in genomic selection (GS) [20] or marker-assisted recurrent selection (MARS) [21]. Li et al. [15] used simple sequence repeat (SSR) markers to transfer a quantitative trait locus (QTL) on chromosome 4D from a synthetic parent, Syn769 to Chuanmai-42. The QTL increased tiller number per plant, number of effective spikes, grains per square meter, harvest index, and grain yield. The authors reported that the average increased grain yield due to this QTL was 8.90%. Additionally, Zhang et al. [5] studied the genetic variation of SYNs and SBLs using SSR markers and concluded that the novel alleles from SYNs were stably inherited in SBL families and introduced the genetic diversity from *Ae*. *tauschii* and durum parents to SBLs. They argued that SYNs and SBLs are valuable genetic resources for broadening genetic diversity of wheat breeding germplasm.

The objectives of this study were i) to determine the capability of SYN lines to increase the genetic diversity of cultivated parents ii) to estimate breeding values of SYN lines and bread wheat parents under fully irrigated, heat and drought stress environments, and iii) to evaluate the performance and estimate breeding values of SDLs in fully irrigated, heat and drought stress environments.

## Materials and Methods

### Population development

The populations of SDLs were developed by crossing 20 CIMMYT spring bread wheat (BW) cultivars to 33 primary SYN lines (S1 Table) using a direct cross (biparental), a first backcross (BC1) and a three-way cross (TC) in 2008. Plants in the segregating populations were selected in a shuttle-breeding program alternating between Yaqui Valley, Ciudad Obregon, north-western Mexico (elevation 38 m, 27°25′ N, 109°54′ W, 320 mm rainfall) and El Batán in the semiarid, subtropical highlands of central Mexico (elevation 2240 m and 19.32°N, 98.51°W, 625 mm rainfall). In the F_1_ generation, individuals of some crosses were selected to create biparental families and some of them were crossed to a recurrent BW parent to create BC_1_ families as part of routine pre-breeding activities to introgress novel genetic diversity into adapted bread wheat backgrounds. Others were crossed to another BW parent to develop TC families. The breeding scheme thereafter was a modified single seed descent in which 50 individual plants (spikes) per cross were selected in the F_2_ generation to plant in F_3_ rows (spike to row). In the F_3_ generation, a single spike per row was selected for the next generation (50 spikes from 50 rows). In the F_4_ and BC_1_F_3_ generations, rows were bulk harvested separately for the next year. F_4:5_ and BC_1_F_3:4_ bulks were planted in 3m long by 80cm wide raised beds and irrigated to increase seed (bed–channel system) and each family had 50 rows. In the early generations, plants were selected that had semi-dwarf plant height and phenology similar to the adapted parents and in the later generations (F_4:5_ and BC_1_F_3:4_), lines were selected for lodging resistance and free threshing. The overall population comprised 97 families with 50 derived F_4:5_ and BC_1_F_3:4_ lines. The number of lines per family was reduced in the F_4:6_ and BC_1_F_3:5_ due to selection for basic agronomic type and uniformity and family sizes ranged from 1 to 48 and the total number of lines was 2080 in the first year yield trials. In the second and third years the number of families was reduced due to selection for easy threshing, early maturity, plant height, and lodging resulting in 80 families consisting of 13 BW parents and 30 SYN parents. The SYN parents were genotyped but were not planted in the field because of the poor agronomic characteristics and lack of threshability.

### Field trials

The selected populations were planted in three parallel trials under the fully irrigated, drought and heat stress conditions at the Norman E. Borlaug Research station (CENEB) in the Yaqui Valley, Ciudad Obregon, northern Mexico (elevation 38 m, 27°25′ N, 109°54′ W) in the year 2011–12. This station is located in an arid region with average precipitation of 320 mm, a mean annual temperature of 24°C, and its soil was a Hyposodic Vertisol (Calcaric, Chromic)[22].

The experimental design, for all trials, was a partially replicated design in which 20 percent of genotypes had two replicates and the remainder was unreplicated. The number of unique genotypes including SDLs, BW parents, and checks in irrigated, drought and heat trials was 2052, 1493, and 1463, respectively, and Vorobey and Quaiu were checks in all trials. The proportions of BC and TC SDLs were 92 and 8%, respectively, for drought and heat trials while for the irrigated trial the BC, biparental, and TC were 68, 27, and 5%, respectively.

The sowing system was bed-channel for the irrigated and heat trials in which each bed (plot) was 3 m long and had two rows 40cm apart with 40cm between beds. Two beds were used for each genotype in the irrigated trial while in the heat trial there was one bed per line. These two trials were fully irrigated. The irrigated trial was planted on December 5^th^, 2011 while the heat trial was planted on March 23^rd^, 2012 to coincide with high temperature stress. The drought trial was planted on December 8^th^, 2011 on a flat plot area without beds and irrigated twice with a drip irrigation system, once at sowing, and again about 45 days later to impose post anthesis drought stress. Plots in the drought trial were wider than the bed system to reduce the relative contribution of plants growing on plot edges and to have a canopy more like in a farmer’s field in a drought stressed growing region. Each plot was 1.6 m wide, 3 m long and had 6 rows.

For the second year, 2012–13, the number of lines was decreased based on grain yield in the irrigated, heat, and drought trials, easy threshing, early maturity, plant height, and lodging. Consequently, the number of unique genotypes including SDLs, BW parents, and checks were 1057, 1054, and 1045 in the irrigated, drought, and heat trials, respectively. These were planted in three parallel trials; fully irrigated, drought, and heat stress, respectively. The sizes of beds and plots were the same as in 2011–12 except for the irrigated trial in which one bed was used per line. Planting dates of the irrigated and heat trials were November 25^th^, 2012 and March 8^th^, 2013. The irrigated and heat trials were irrigated five and six times through gravity flood-irrigation, respectively. The drought trial was irrigated twice.

In the year 2013–14, the irrigated, drought and heat trials were planted on December 6^th^, 2013, December 20, 2013, and February 27^th^, 2014, respectively. The irrigation system and number of irrigations of trials were the same as the second year. Also, the unique number of lines in the irrigated, heat and drought trials was 1056, 1056, and 1054, respectively.

Field experimental design for heat and irrigated trials in the years 2012–13 and 2013–14 was alpha lattice with two replicates while for drought trials it was augmented design. The cultivars Vorobey, Navojoa, Roelfs, Reedling and Quaiu were checks in all trials. The BC and biparental SDLs made up the main part of the population with proportions of 74% and 20%, respectively, followed by 6% TC populations.

### Phenotyping

Each year, plant height (PLH), days to heading (DHE), days to flowering (DFL), days to maturity (DMA), and grain yield (YLD t/ha) were measured in all trials according to Pask et al. [23]. Thousand kernel weight (TKW) and grain filling duration (GFD) were only measured for the irrigated trial in the year 2011–12 [23].

#### Phenotypic data analysis

The experimental designs were different for each year and trial complicating combined analysis of all trials. To correct for within field heterogeneities spatial analysis was used for each trait/trial combination separately based on row and column orders. The Genstat software [24] was used for analysis of the general linear mixed model by the following equation;

$$Y=X\beta +Z_{R}u_{R}+Z_{C}u_{C}+\varepsilon$$

where ***Y*** is the response vector, **X** is a design matrix for fixed effects such as overall mean and genotype effects. ***Z***_***R***_ is a design matrix for row effects, ***Z***_***C***_ is a design matrix for column effects, ***β*** is a vector for fixed effects, ***u***_***R***_ and ***u***_***C***_ are vectors for random row and column effects with $u_{R}\;\sim \;N\left(0,\sigma_{R}^{2}\;I\right),$ and $u_{C}\;\sim \;N\left(0,\sigma_{C}^{2}\;I\right)$ correspondingly and ***ε*** is a residual vector with $\varepsilon \;\sim \;N\left(0,\;\sigma_{R}^{2}R\right),$ where R is given by $R=Z_{\varepsilon }\left[AR1\left(\rho_{R}\right)⊗AR1\left(\rho_{C}\right)\right]Z_{\varepsilon }^{\prime }$. *AR*1(*ρ*_*R*_) is an auto-regressive order one correlation matrix for row effects, *AR*1(*ρ*_*C*_) is an auto-regressive order one correlation matrix for column effects and *Zε* is a design matrix for row and column combinations. Consequently, row and column effects were removed in each trial and best linear unbiased estimates (BLUEs) of genotypes were generated for subsequent analysis.

Pearson correlation was used to estimate the phenotype correlation coefficients among environments for all traits.

### Genotyping

Genomic DNA was extracted from dried leaves collected from a single plant for each line using a modified CTAB (cetyltrimethylammonium bromide) method[25] modified as shown in CIMMYT laboratory protocols [26] and quantified using NanoDrop 8000 spectrophotometer V 2.1.0. The genotyping of the samples was accomplished using a genotyping-by-sequencing technique called DArTseq^™^ developed by DArT Pty. Ltd., Yarralumla, Australia. The detailed protocol is described in Sehgal et al.[27]. A total of 20,468 genotyping–by–sequencing (GBS) markers were used for genotyping of 1991 lines. Marker data were filtered for missing data (< 50%) and minor allele frequency (MAF) (< 1%) for a final number of 10,262 GBS markers selected for subsequent analysis.

#### Kinship matrices

The genomic relationship matrix, **G** matrix, was generated using 10,262 GBS markers. The rrBLUP package in R [28] was used to impute the missing data based on expectation maximization (EM) imputation algorithm and generate the **G** matrix.

The numerator relationship matrix, **A** matrix, was created based on pedigree information for populations that included 1986 individuals. More specifically, to generate the **A** matrix, we compared the relatedness of parents and different crosses; biparental, BC and TC for SDLs. For relatedness of SYN lines, *f* = 0.66 if they had the same durum parents but a different *Ae*. *squarrosa* parent and *f =* 0.33 if they had the same *Ae*. *squarrosa* parent but a different durum parent. For some SYN lines *f* = 1 if they had the same durum and diploid parents. For BW parents, most of them were unrelated except for two pairs that were identical and *f* = 1 was used for them.

The heat map of the **G** matrix indicated that there could be some individuals with inconsistencies between the familial relationships given by the **A** matrix and the relationships indicated by the **G** matrix. These individuals were designated as outlier individuals and removed from further study. More specifically, to identify the potential outlier individuals in each family, a distance matrix was created using imputed marker data. Individuals with a distance larger than Q3+1.5(IQR), where Inter-Quartile Range (IQR) = Q3−Q1, Q1 is the 25^th^ percentile and Q3 is the 75^th^ percentile, within each family were considered outliers. Consequently, 144 individuals belonging to 72 families (from 1 to 7 individuals) were removed from further study. This resulted in the correlation coefficient between off diagonal elements of **A** and **G** matrices increasing from 0.65 to 0.75. Therefore, 1846 genotyped individuals were used for subsequent analyses.

The **H** matrix is a pedigree-marker relationship matrix that modifies the genetic relationship matrix to combine pedigree-based relationship information [29–31]. In this study, the **H** matrix was used to combine the pedigree information of 1986 lines with the marker information of 1846 lines. The following covariance matrix was used to create the **H** matrix;

$$H=[\begin{array}{cc} A_{11}+A_{12}A_{22}^{-1}(G_{w}-A_{22})A_{22}^{-1}A_{21} & A_{12}A_{22}^{-1}G_{w}\\ G_{w}A_{22}^{-1}A_{21} & G_{w} \end{array}]$$

where the pedigree-based relationship matrices **A**_**11**_ and **A**_**22**_ are sub-matrices of **A** matrix for genotyped and non-genotyped individuals, respectively, and **A**_**12**_ or **A**_**21**_ is the covariance matrix between genotyped and non-genotyped individuals. **G**_**w**_ is the weighted **G** matrix, **G**_**w**_ = w*G + (1-*w*)*A_**22**_, **G** is the genomic relationship matrix and ***w*** is the weight for contribution ratio of **A** matrix or portion of genetic variance that was not explained by markers. The ranges of ***w*** were from 0 to 1 by 0.1 interval, ***w*** = 1 represents the **G** matrix and **w =** 0 indicates **A** matrix. In this study different values of ***w*** were used to create the **H** matrix and ***w*** = 0.1 gave the best overall results in terms of prediction accuracies in the validation data. Hence, ***w*** = 0.1 was used to create the **H** matrix, which included 1986 genotyped and non-genotyped individuals.

#### Genomic estimated breeding values

The genomic best linear unbiased prediction (GBLUP) model was used to estimate both variance components and genomic estimated breeding values (GEBVs). All analyses were executed with the EMMREML package in R software [32]. BLUPs were computed using the following univariate mixed model:

y=Xβ+Zu+ϵ

where ***y*** is a vector of spatially corrected observations of genotyped individuals for the traits of interest, **X** is a known design matrix for fixed effects which comprised management (Irrigated, heat, and drought environments) and year, **Z** is a known design matrix for random effects (individuals), **β** is a vector for non-genetic fixed effects, **u** is a vector for genetic random effects or breeding values with $u\;\sim \;N\left(0,\;\sigma_{u}^{2}G\right),$ **G** is the genomic relationship matrix and ***ϵ*** is a residual vector with $\epsilon \;\sim \;N\left(0,\;\sigma_{e}^{2}I_{n}\right)$ [33]. Breeding values were then estimated by solving the mixed model equations. The same model was also fitted by replacing the **G** matrix with **A** and **H** matrices.

#### Cross Validation and Genomic prediction

The 5-fold cross validation was used to quantify the fidelity of genomic prediction of traits for each trial and all trials together [34]. The accuracy of estimates was based on the correlation between *y − Xβ* and GEBVs. The marker, pedigree and pedigree–marker models were used in the training set based on the GBLUP method as described above. Also, mean heritability of traits was estimated using $\frac{\sigma_{u}^{2}}{\sigma_{u}^{2}+\left(\frac{\sigma_{e}^{2}}{r}\right)}$ in which $\sigma_{u}^{2}$ and $\sigma_{e}^{2}$ are genetic and error variances, respectively, and *r* is the number of replicates for each individual.

#### Genetic diversity

To measure genetic diversity of BWs, SYNs, SDLs, Nei’s gene diversity, *Hs*, was used [35]. There were 8,612 out of 10,262 SNPs, that had chromosome information, and those were filtered for missing data (NA < 10%) within each group of BWs, SYNs and SDL populations.

The hierarchical cluster analysis with the Ward method and Euclidean distance [36] was used to classify the BW and SYN parents based on whole genome marker information, 10,262 SNPs.

## Results

### Phenotypic analysis

The summary information for traits from each trial and year is presented in Table 1. Means of the traits in the irrigated trials were similar across the years while means of traits varied widely in the heat and drought trials. For example, DRO.Y13.14 had the lowest mean value, especially for YLD (1.054 t/h), HEAT.Y11.12 had the lowest mean values for PLH and YLD and differed greatly from those in the other two heat trials. This was caused by late planting resulting in very low yield with some genotypes not producing any grain. For this year, YLD ranged from 0 to 2.40 t/h and PLH ranged from 20 to 70 cm. Thus, it was considered to be an outlier environment and the data were not used in subsequent analyses (Table 1).

**Table 1:** Mean and range of traits in different trials in years 2011–14 in Ciudad Obregon, CIMMYT, Mexico.

Trial\Trait	DHE	DFL	DMA	PLH (cm)	YLD (t/h)
IRRI.Y11.12	-	81^a^ (61–95)^b^	128 (119–36)	114 (87–150)	6.34 (2.90–8.50)
IRRI.Y12.13	73 (58–93)	78 (63–97)	126 (117–36)	102(82–121)	5.95 (2.78–8.94)
IRRI.Y13.14	75 (65–88)	79 (69–92)	121 (107–33)	102 (86–121)	5.55 (3.18–7.59)
DRO.Y11.12	-	81 (72–99)	117 (104–30)	84 (58–120)	2.42 (1.09–3.56)
DRO.Y12.13	75 (65–87)	78 (66–92)	-	-	2.30 (1.55–2.95)
DRO.Y13.14	67 (58–79)	69 (60–80)	100 (91–109)	70 (50–96)	1.05 (0.49–1.40)
HEAT.Y11.12	-	-	-	42 (20–70)	0.57 (0.00–2.40)
HEAT.Y12.13	50 (45–59)	-	81 (78–89)	61 (45–75)	1.96 (0.29–3.18)
HEAT.Y13.14	56 (50–66)	59 (54–69)	87 (82–96)	59 (41–89)	2.07 (0.33–3.26)

DHE: Days to heading, DFL: Days to flowering, DMA: Days to maturity, PLH: Plant height, and YLD: Grain Yield t/h. IRRI: Irrigated, DRO: Drought, HEAT: Heat trials, Y11.12: Year 2011–12, Y12.13: Year 2012–13, and Y13.14: Year 2013–14 (e.g. IRRI.Y11.12: irrigated trial in the year 2011–12).

^a^; Mean of the trait,

^b^; Range of the trait.

All phenotypic correlation coefficients among environments for PLH and YLD were significant (Table 2). For YLD, correlations within treatments (irrigated, heat or drought) across the three years ranged from 0.54 to 0.60 for irrigated trials, 0.42 to 0.61 for heat trials, and 0.42 to 0.49 for drought trials while, correlations between different treatments ranged from 0.13 to 0.59. Over all the trials, correlation coefficients for YLD ranged from 0.13 to 0.61 for HEAT.Y11.12 with IRRI.Y12.13 and HEAT.Y11.12 with HEAT.Y13.14, respectively (Table 2 below diagonal). For PLH, correlations within treatments across the three years ranged from 0.68 to 0.78 for irrigated trials, 0.38 to 0.50 for heat trials, and 0.52 for drought trials while, correlations between different treatments ranged from 0.33 to 0.65. Among treatments, correlations for PLH ranged from 0.33 to 0.65 for HEAT.Y11.12 with DRO.Y13-14 and IRRI.Y11.12 with DRO.Y12.13, respectively (Table 2 above diagonal).

**Table 2:** Phenotypic correlations for PLH (above diagonal) and YLD (below diagonal) within and among environments.

Trial/Trait	PLH								
IRRI.Y11.12	1	0.78*	0.69	0.65	-	0.47	0.43	0.44	0.58
IRRI.Y12.13	0.54	1	0.68	0.55	-	0.41	0.41	0.44	0.56
IRRI.Y13.14	0.60	0.54	1	0.58	-	0.43	0.36	0.39	0.56
DRO.Y11.12	0.36	0.14	0.22	1	-	0.52	0.35	0.39	0.51
DRO.Y12.13	0.39	0.34	0.42	0.48	1	-	-	-	-
DRO.Y13.14	0.27	0.17	0.26	0.42	0.49	1	0.33	0.36	0.51
HEAT.Y11.12	0.35	0.13	0.20	0.33	0.33	0.26	1	0.38	0.45
HEAT.Y12.13	0.45	0.41	0.45	0.38	0.59	0.40	0.42	1	0.50
HEAT.Y13.14	0.38	0.28	0.35	0.31	0.52	0.44	0.61	0.59	1
**YLD**	IRRI. Y11.12	IRRI. Y12.13	IRRI. Y13.14	DRO. Y11.12	DRO. Y12.13	DRO. Y13.14	HEAT. Y11.12	HEAT. Y12.13	HEAT Y13.14

IRRI: Irrigated, DRO: Drought, HEAT: Heat trials, Y11.12: Year 2011–12, Y12.13: Year 2012–13, and Y13.14: Year 2013–14.

*: All correlation coefficients were significant.

Phenotypic correlations for DFL (Table 3 below diagonal), DMA (Table 3 above diagonal), and DHE (Table 4) were significant and ranged from 0.26 to 0.84. For these traits, correlations between and within trials for the three years were medium to high except for some low correlations observed for DMA between HEAT.Y12.13 with IRRI.Y11.12 and HEAT.Y12.13 with DRO.Y11.12 (Table 3 above diagonal).

**Table 3:** Phenotypic correlation for DMA (above diagonal) and DFL (below diagonal) within and among environments.

Trial/Trait	DMA							
IRRI.Y11.12	1	0.56*	0.56	0.54	-	0.58	0.26	0.40
IRRI.Y12.13	0.70	1	0.58	0.50	-	0.71	0.36	0.59
IRRI.Y13.14	0.73	0.82	1	0.48	-	0.62	0.35	0.41
DRO.Y11.12	0.61	0.51	0.56	1	-	0.62	0.28	0.38
DRO.Y12.13	0.51	0.50	0.52	0.44	1	-	-	-
DRO.Y13.14	0.75	0.84	0.81	0.57	0.55	1	0.40	0.58
HEAT.Y12.13	-	-	-	-	-	-	1	0.46
HEAT.Y13.14	0.54	0.72	0.62	0.39	0.36	0.68	-	1
**DFL**	IRRI. Y11.12	IRRI. Y12.13	IRRI.Y13.14	DRO. Y11.12	DRO. Y12.13	DRO. Y13.14	HEAT. Y12.13	HEAT. Y13.14

*: All correlation coefficients were significant.

**Table 4:** Phenotypic correlation for DHE within and among environments.

Trial/Trait	DHE					
IRRI.Y12.13	1					
IRRI.Y13.14	0.82*	1				
DRO.Y12.13	0.54	0.59	1			
DRO.Y13.14	0.84	0.82	0.62	1		
HEAT.Y12.13	0.44	0.42	0.31	0.48	1	
HEAT.Y13.14	0.73	0.63	0.41	0.68	0.54	1

*: All correlation coefficients were significant.

The range for TKW for the IRRI.Y11.12 trial was from 40 to 65 gr for SDL populations while for 13 BW parents the range was from 41 to 54 gr and for the top 10% of the populations (the top 10% was based on YLD) it was 41 to 58 gr (S2 Table). Sixty seven percent of SDLs had higher TKW than their corresponding recurrent BW parents. Furthermore, among 26 biparental families, the TKW mean decreased by -2 to -3.92% for four populations, while it increased from 0.67 to 24.39% for 22 populations compared to the TKW mean of the BW parents. The same comparison for 38 BC populations showed that TKW of six populations decreased by– 0.44 to -5.40% while TKW for 32 of them increased from 3.3 to 16.1%. Among the four TC populations, one had the highest reduction for TKW (-17.9%) but TKW for the other three populations increased from 6.83 to 12.68% (S2 Table).

The range of GFD was from 48 to 62 days over all genotypes in the IRRI.Y11.12 trial. For the 13 BW parents it ranged from 49 to 60 days and for the top 10% of the SDL populations it ranged from 48 to 61 days (S2 Table).

Relationships between TKW and GFD were significantly positive over the all populations (*y* = 0.21*x* + 44; *P* < 0.001, *R*^2^ = 0.17) and for the top 10% of the SDL populations (*y* = 0.15*x* + 46; *P* < 0.001, *R*^2^ = 0.05) in the IRRI.Y11.12 trial.

Relationships between YLD and GFD were significantly negative over all populations (*y* = −0.032*x* + 8.40; *P* < 0.001, *R*^2^ = 0.02) while it was not significant for the top 10% of the SDL populations (*y* = −0.022*x* + 8.40, *R*^2^ = 0.009). Also, significant a negative relationship was observed between YLD and TKW overall and for the top 10% of the SDL populations (*y* = −0.017*x* + 7.50; *P*<0.001, *R*^2^ = 0.02) and (*y* = −0.017*x* + 8; *P* < 0.05, *R*^2^ = 0.08), respectively.

### Clustering of bread wheat and synthetic parents

**Fig 1:**
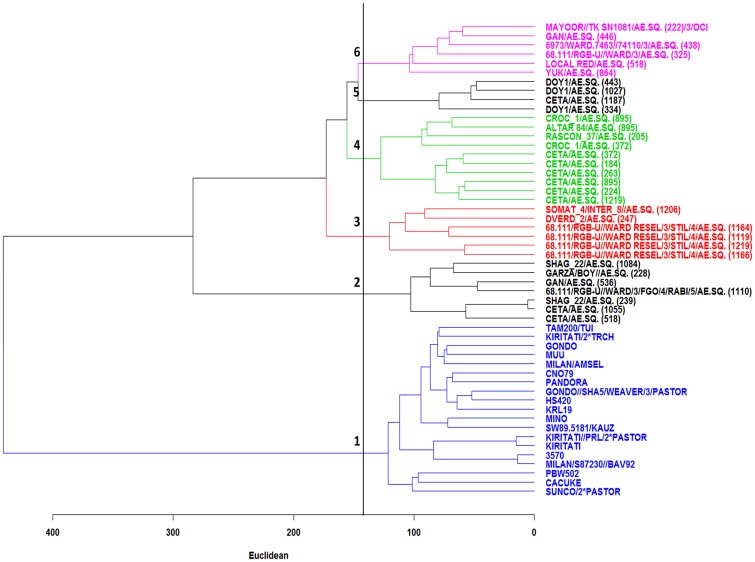
Dendrogram of the classification of BW parents (Blue color) and SYN lines using the Ward method based on polymorphic SNP markers.

As expected, the dendrogram of the hierarchal cluster analysis revealed that SYN lines were more genetically diverse than BW parents (Fig 1). For instance, using an arbitrarily cut off, BW parents made one group, cluster 1, while SYN lines grouped into five different clusters.

**Fig 2:**
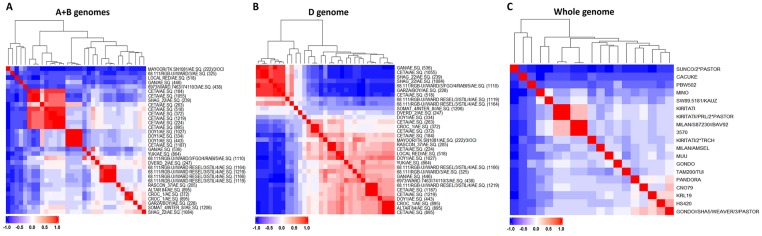
Heat map for SYN and BW parents based on genome-specific marker information. (A) Clustering of SYN parents using A+B genomes and (B) D genome, (C) Clustering of BW parents based on whole genome.

Marker information for A+B and D genomes were used to investigate the genetic diversity of durum (Fig 2A) and *Ae*. *squarrosa* parents (Fig 2B) that were used to develop SYN parents. Seventeen durum parents were grouped into four clusters. Cluster 1 comprised five unrelated durum parents. Cluster 2 had only two durum parents CETA and SHAG_22 crossed to AE.SQUARROSA 239, however the durum parent named SHAG_22 was likely to be CETA. Cluster 3 had two durum parents DOY1 and CETA crossed to AE.SQUARROSA 1187, however the durum parent named CETA was likely to be DOY1. Cluster 4 comprised 11 unrelated durum parents (Fig 2A).

Based on D genome markers, 28 AE.SQUARROSA parents were grouped into three clusters. Cluster 1 included seven AE.SQUARROSA that were closely related (Fig 2B). Cluster 2 comprised four unrelated AE.SQUARROSA parents. Cluster 3 included 22 AE.SQUARROSA parents in which some of them were highly related or identical.

Based on whole genome marker information, most of the BW parents of this study were not closely related except for two pairs of lines (Fig 2C). For KIRITATI and KIRITATI//PRL/2*PASTOR BW parents, this could have resulted from being sister lines or from selfed progenies of KIRITATI. For MILAN/S87230//BAV92 with BW line 3570, an error in labeling or seed packaging is more likely. Errors in pedigrees will affect predictions when using the pedigree based relationship **A** matrix or **H** matrix. However, we corrected these errors when generating the **A** matrix.

Genome distribution of the markers and Nei’s genetic diversity (*Hs*) for each genome for BW, SYN parents, and SDLs are shown in Table 5. SNP markers were not evenly distributed in the three genomes. The D genome with 3691 had the most markers and the A genome with 2333 had the lowest. For SYNs, *Hs* for A, B, and D genomes were 0.35, 0.38, and 0.40, respectively, and they were greater than those for the BW parents, which were 0.27, 0.26, 0.06 (Table 5). For SDLs, *Hs* was 0.36 for A and B genomes and 0.19 for the D genome, all greater than those for BW parents. The mean genetic diversity was 0.19 for BWs, 0.38 for SYNs and 0.28 for SDLs (Table 5).

**Table 5:** Distribution of markers and diversity index (*Hs*) in each genome for BWs, SYNs and SDLs.

		No. marker after filtering for NA < 10%			*H_s_*		
Genome	No. marker	BWs	SYNs	SDLs	BWs	SYNs	SDLs
A	2333	1443	404	1595	0.27	0.35	0.36
B	2587	1584	468	1747	0.28	0.38	0.36
D	3691	2073	929	2630	0.06	0.40	0.19
Total/Mean	8612	5100	1801	5972	0.19	0.38	0.28

### Estimating genomic breeding value of parents

#### Cultivated wheat parents

**Fig 3:**
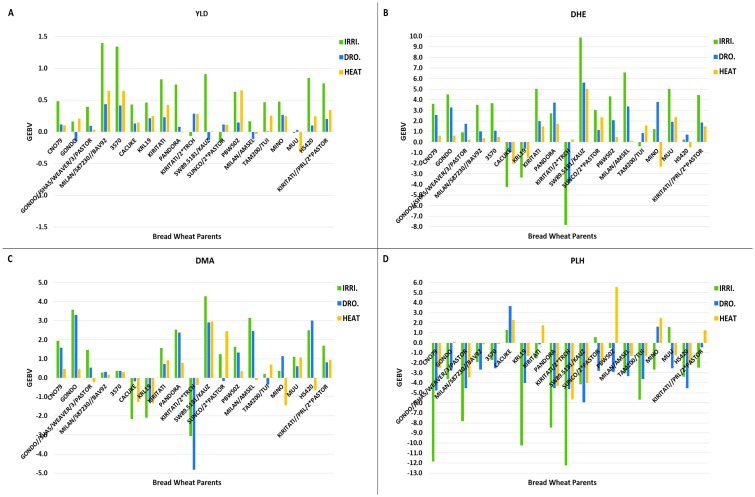
GEBVs of BW parents for traits in three contrasting environments. Irrigated (IRRI.), Drought (DRO.), and Heat (HEAT): (A) grain yield (YLD) GEBVs, (B) days to heading (DHE) GEBVs, (C) days to maturity (DMA) GEBVs and (D) plant height (PLH) GEBVs across three environments.

Most of the BW parents had positive GEBVs for grain yield across all environments and their values ranged from -0.16 to 1.40 under irrigated, -0.15 to 0.43 under drought, and -0.33 to 0.65 under heat environments (Fig 3A and S3 Table). Among BW parents, MILAN/S87230//BAV92 and BW line 3570 were the best parents and had the highest GEBVs across three environments while MUU, SUNCO/2*PASTOR and MILAN/AMSEL were the poorest parents with very small positive values in one environment and negative values in the other environments. Parents reflected genotype by environment interaction (GEI) and they usually had the highest GEBVs in the irrigated trials except for KIRITATI/2*TRCH, SUNCO/2*PASTOR, and MUU that had negative values. Generally, GEBVs of parents decreased in stress conditions except for PBW502 and GONDO that had almost the same positive value in irrigated and heat environments. However, SUNCO/2*PASTOR and KIRITATI/2*TRCH had negative yield GEBVs in the irrigated trials and positive values in the heat and drought stress trials.

For DHE, almost all the BW parents had positive GEBVs across environments except KIRITATI/2*TRCH, CACUKE, KRL19. The GEBVs ranged from -7.88 to 9.88 for irrigated, from -3.89 to 5.61 for drought, and from -2.33 to 5.05 for heat environments. SW89.5181/KAUZ had the highest positive GEBVs across all environments while KIRITATI/2*TRCH had the highest negative GEBVs under irrigated and drought conditions. For this trait, GEI was observed and HS420 had very low GEI across environments (Fig 3B and S3 Table).

For DMA, the trend for GEBVs of BW parents was similar to those for DHE but the values decreased for all parents except for HS420 which increased in drought and irrigated conditions (Fig 3C and S3 Table). Also, MILAN/S87230//BAV92 and BW line 3570 showed less GEI for DMA than for DHE.

The PLH GEBVs were nearly all negative for BW parents except for CACUKE that had positive values in all environments and four other parents that had at least one positive value in one environment (Fig 3D and S3 Table).

#### The GEBV values of synthetic lines

**Fig 4:**
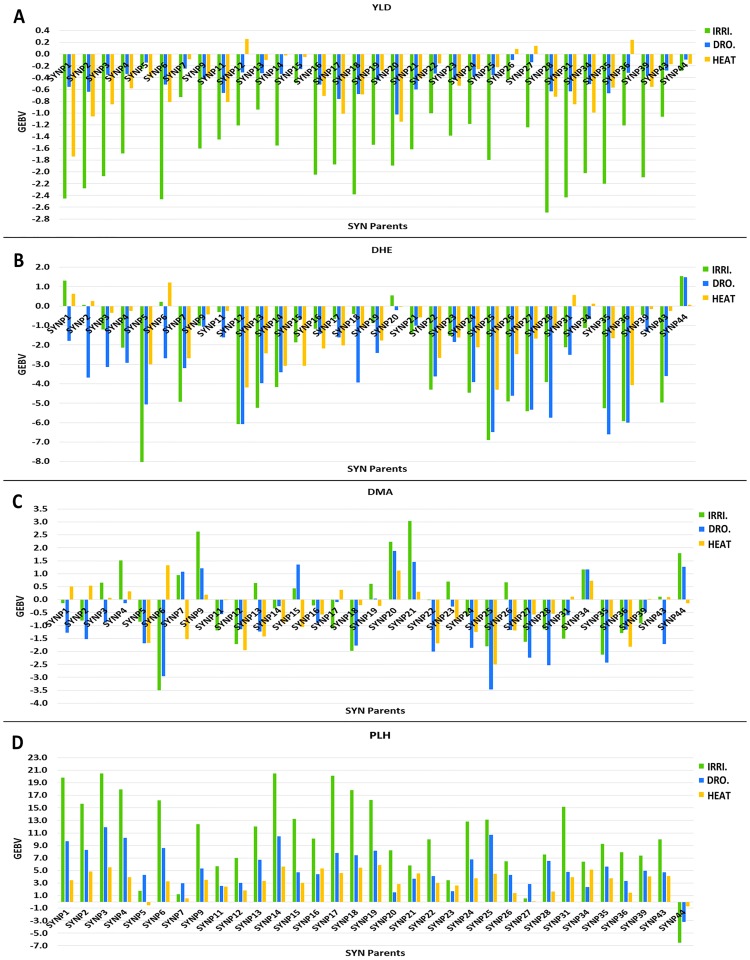
GEBVs of SYN parents for traits in three contrasting environments. Irrigated (IRRI.), Drought (DRO.), and Heat: (A) grain yield (YLD) GEBVs, (B) days to heading (DHE) GEBVs, (C) days to maturity (DMA) GEBVs and (D) plant height (PLH) GEBVs across three environments.

All of the SYN lines had negative GEBVs for grain yield across all environments except SYNP12, SYNP26, SYNP27, and SYNP36 that had small positive values under the heat stress. Predominantly, they had the lowest GEBVs in irrigated condition (-0.25 to -2.69) while their value ranged from -0.10 to -1.02 for drought and from 0.26 to -1.74 for heat stress (Fig 4A and S4 Table). However, these results were expected, because SYN lines have very low grain yield.

For DHE, GEBVs of all SYN lines were negative and decreased DHE except for six SYN parents that had positive values in all or at least one environment (Fig 4B and S4 Table). GEBVs for DHE ranged from -8.04 to 1.55 under irrigated, -6.61 to 1.48 under drought and -4.31 to 1.21 under heat conditions (S4 Table). Most of the SYN lines had less strongly negative GEBVs under heat stress indicating that they strongly influenced them to head earlier. For DFL, most of the SYN lines showed similar trends across all environments (S4 Table).

For DMA, breeding values of SYN parents were more variable than those for DHE and many parents had positive GEBVs in one or more environments (Fig 4C and S4 Table). Also, SYN parents had overall lower negative GEBVs for DMA than DHE and increased DMA. Under irrigated environments, the range of GEBVs was -3.50 to 3.04, -3.46 to 1.87 for drought and -2.5 to 1.33 for heat stress trials. GEI for DMA was greater than that for DHE.

All SYN lines contributed to increased PLH in all environments except SYNP44, which had negative GEBVs. Their GEBVs were higher in irrigated trials and ranged from -6.53 to 20.51 while they had lower values in heat stress trials ranging from -0.55 to 5.84 (Fig 4D and S4 Table).

#### Correlation of parent GEBV values across environments

All GEBV correlation coefficients among environments for BW parents were significant (Table 6 above diagonal). The correlations between GEBVs for drought stress and those for irrigated environments were lower than those between irrigated and heat, and drought and heat environments. For SYN lines, correlations between different environments were significant (Table 6, below diagonal) and they showed lower GEI than BW parents.

**Table 6:** Pearson correlation coefficients of parent GEBVs across environments for yield.

	**BW parents**		
Environments	IRRI.	DRO.	HEAT
IRRI.	1	0.47	0.62
DRO.	0.73	1	0.68
HEAT	0.70	0.72	1
**SYN parents**			

IRRI.: Irrigated, DRO.: Drought and HEAT: Heat.

### Performance of synthetic-derived lines in different environments

**Fig 5:**
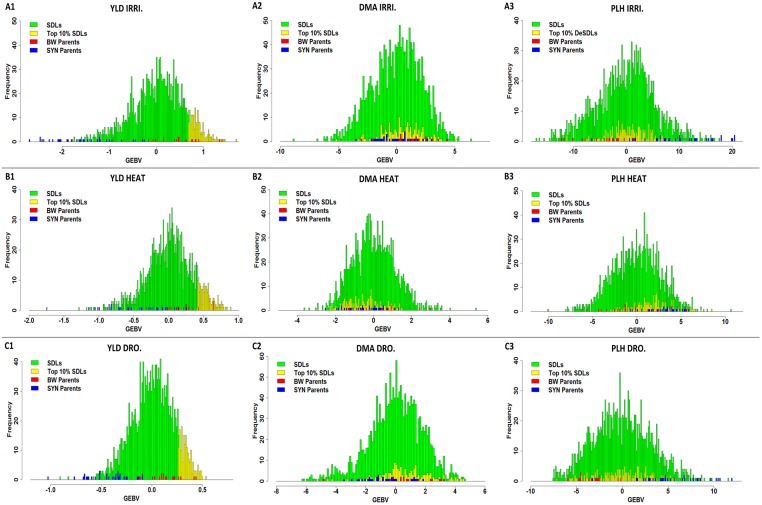
Distribution of GEBVs for the SDLs, SYN and BW parents in different trials. Figure 5 compares the top 10% of SDLs (yellow tail) to BW and SYN parents that are constant in each trial for three traits (YLD, DMA and PLH): (A1) distribution of YLD GEBVs in irrigated trials, (A2) distribution of DMA GEBVs in irrigated trials in which, GEBVs of the top 10% SDLs are in the same range of the parents, (A3) distribution of PLH’s GEBVs in irrigated trials in which PLH of the top 10% SDLs were skewed toward the BW parents, (B1) distribution of YLD GEBVs in heat trials, (B2) DMA GEBVs in heat trials where GEBVs of the top 10% SDLs were placed within the range of GEBVs of the parents. (B3) distribution of PLH GEBVs in heat trials. GEBVs of the top 10% SDLs were skewed toward the SYN parents, (C1), (C2) and (C3) are for drought trials.

Crossing SYN lines to BW parents extended their genetic diversity for measured traits. The variation for grain yield GEBVs was greatest under irrigation and ranged from –2.02 to 1.69 for SDLs, while it ranged from -0.16 to 1.34 for BWs (Fig 5A1). Variation in yield GEBVs was least under drought stress ranging from -0.91 to 0.54 for SDLs and from -0.15 to 0.43 for BWs (Fig 5C1). Under heat stress, GEBV variation ranged from -1.28 to 0.88 for SDLs and -0.326 to 0.649 for BWs (Fig 5B1).

To determine how many SYN parents were able to improve the YLD of BW parents in different environments, the top 10% of SDLs was selected and the average GEBV values for each cross or family was compared to their corresponding recurrent BW parent’s GEBV values (Fig 5). This top 10% included progenies of 13 BW and 23 SYN parents in which MILAN/S87230//BAV92, SUNCO/2*PASTOR, PANDORA, SYNP4, SYNP5, SYNP17, SYNP20, SYNP21, SYNP23, SYNP27, SYNP39, and SYNP43 had major contributions across all environments (S5–S7 Tables).

#### Heat stress

The top 10% SDLs in heat stress comprised 175 SDLs and the average GEBVs of SDLs in each cross was higher than those of their corresponding recurrent BW parents except for SDLs in crosses with MILAN/S87230//BAV92, PBW502, and BW line 3570 (S5 Table). The increased GEBVs for SDLs compared to their BW parents ranged from 2 to 427% and included mainly BC progenies. However, there was also one TC and six biparental crosses in which the progenies had higher GEBVs than the BW parent (S5 Table). Under heat stress the average yield GEBVs of the top 10% of SDLs was 0.55 while the weighted average GEBV of their recurrent BW parents was 0.26 (Fig 5B1).

#### Irrigated environment

In the irrigated trials, the average yield GEBVs of the top 10% of SDLs ranged from 0.69 to 1.09 while these values ranged from -0.16 to 1.40 for BW parents (S6 Table). The average GEBVs of SDLs of crosses with BW line 3570, HS420, MILAN/S87230//BAV92, KIRITATI, PANDORA (in 3 crosses), and SW89.5181/KAUZ (in 1 cross) decreased by -2 to -49% while GEBVs of SDLs of crosses with other BW parents increased by 8 to 111%. In the irrigated trials, BC progenies had generally higher GEBVs but there was one TC and three biparental crosses whose progenies had higher GEBVs (S6 Table). The average GEBV of the top 10% of SDLs was 0.94 while the weighted average GEBV was 0.90 for their recurrent BW parents (Fig 5A1).

#### Drought stress

The top 10% of the SDLs of populations grown under drought stress involved 179 SDLs for which the average GEBVs of crosses ranged from 0.26 to 0.44 while the range for BW parents was -0.11 to 0.44. The increased GEBVs for SDLs compared to the corresponding recurrent BW parents ranged from 12 to 422% (S7 Table). However, the cross of SYN parents to MILAN/S87230//BAV92 did not improve the GEBVs of its SDLs. Also, the average yield GEBV of the top 10% of SDLs was 0.34 while the weighted average GEBV was 0.30 for their corresponding recurrent BW parents (Fig 5C1).

Across all environments, it was observed that the SYN lines most significantly increased grain yield of low yielding BW parents in both stress and normal conditions (S5–S7 Tables). For example, SUNCO/2*PASTOR was a low-yielding BW parent across all environments. In crosses with SYNs it contributed 59 progenies in the top 10% of SDLs and all of them outperformed the BW parent. Their yield GEBVs ranged from 0.27 to 1.20 under drought stress and irrigated conditions, respectively, while the range of yield GEBVs for SUNCO/2*PASTOR was from -0.16 to 0.12 under irrigated and drought environments, respectively. The high-yielding BW parents, MILAN/S87230//BAV92, had 173 progenies among the top 10% of the SDLs, but only 25 of them had higher GEBVs than the BW parent. Their GEBVs for yield ranged from 0.44 to 1.69 under drought and irrigated conditions, respectively. This pattern is similar for the other low- and high-yielding BW parents.

In order to determine if the high yield of SDLs is related to the phenological traits, the correlation coefficients between GEBVs of YLD and those for other traits was calculated. Squared correlations of YLD with DMA were 0.08, 0.06, and 0.04 in drought, heat and irrigated, respectively, and these values for PLH were 0.01, 0.05, and 0.06 in drought, heat and irrigated, respectively which indicated that DMA and PLH did not affect the yield.

### Genomic Prediction

The univariate, random five-fold cross validation was used for genomic prediction of traits for each trial and for all trials together. As was previously mentioned, the heat trial in year 2011–12 experienced extreme temperatures and when using the phenotypic observations from this trial in genomic prediction models, both heritability and prediction accuracy of the traits decreased across environments. Consequently, this trial was excluded from cross validation.

Broad-sense heritabilities of traits in different environments based on pedigree, marker and pedigree-marker models are shown in Table 7. Estimated heritabilities for all traits using the corrected pedigree model were slightly higher in each environment except for DFL under the heat stress environments (Table 7). The differences in heritabilities could be due to 1) the artificially high genetic variance assigned to unrelated parents that are actually related 2) the differences in the amount of estimated genetic variances using **A** or **G** matrices in the model [37]. We observed that estimated genetic variances using the **G** matrix (gVarG) were smaller than those using the **A** matrix (gVarA) for all traits under drought stress. Under heat stress, gVarG for all traits were smaller than gVarA except for DFL, and under the irrigated environment, the trend was similar except for DFL and PLH. The genetic variances estimated using the **G** matrix explained 66 to 96% (gVarG/ gVarA) of those estimated using the **A** matrix under drought stress. This ratio ranged from 81 to 131% and from 76 to 118% for the heat and irrigated environments, respectively 3) Sampling error due to finite markers can affect the estimation of the **G** matrix as reported by Haile- Mariam et al. [38] and Powell et al [39]. 4) All the diagonal elements of the **A** matrix were 2 while the average of the diagonal elements of the marker based relationship matrix (**G** matrix) was 1.86 (0.25 to 9.99). However, scaling the **G** matrix did not change the results (data not shown).

**Table 7:** Mean heritability for traits in each trial.

Trials	DRO.			IRRI.			HEAT		
Model/Trait	Pedigree	Marker	Pedigree-Marker	Pedigree	Marker	Pedigree-Marker	Pedigree	Marker	Pedigree-Marker
DHE	0.68	0.63	0.64	0.71	0.67	0.67	0.64	0.58	0.59
DFL	0.68	0.64	0.64	0.77	0.75	0.75	0.24	0.34	0.37
DMA	0.70	0.62	0.64	0.69	0.66	0.66	0.54	0.49	0.50
PLH	0.60	0.51	0.53	0.80	0.79	0.79	0.61	0.53	0.56
YLD	0.57	0.52	0.52	0.70	0.64	0.65	0.68	0.63	0.64

The trait heritabilities were consistently higher in irrigated than drought and heat stress environments using the three models. Heritabilities of DHE, DMA and DFL were higher under irrigated and drought environments but lower under heat stress especially for DFL (Table 7). This could be related to the lower number of observations for these traits. DHE and DMA had two years of data but DFL had only one year of data. PLH had the highest heritability under irrigated environments (0.79–0.80) and decreased under drought and heat stress (0.51 to 0.61) (Table 7). Also, the highest heritability for YLD was observed under irrigated (0.64–0.70) followed by heat (0.63–0.68) and drought stresses (0.52–0.57) (Table 7).

Predictability was assessed as the correlation between GEBVs and observed phenotypes and were corrected for fixed effects by cross-validation. Our results showed that the marker model gives higher genetic prediction accuracy than the pedigree model for all traits either in the single environments (e.g. Irrigated, heat, and drought) (Table 8) or combined environments (Table 9). Mean accuracy of the three models ranged from 0.30 to 0.64 across all environments. The highest prediction accuracy was obtained in irrigated environments while lower accuracies were mostly observed in heat stress environments. Increased prediction accuracy using the marker model ranged from 2% for YLD to 5% for DHE under drought stress. This range was 5% for PLH to 9% for DFL under irrigation and 5% for YLD to 12% for PLH and DHE in heat stress. Using the marker-pedigree model did not improve the prediction accuracy (Table 8).

**Table 8:** Mean genomic prediction accuracy of traits for each trial in cross validation.

Trials	DRO.			IRRI.			HEAT		
Model/Trait	Pedigree	Marker	Pedigree-Marker	Pedigree	Marker	Pedigree-Marker	Pedigree	Marker	Pedigree-Marker
DHE	0.49	0.54	0.55	0.54	0.61	0.64	0.33	0.45	0.45
DFL	0.45	0.48	0.50	0.49	0.58	0.60	0.43	0.52	0.55
DMA	0.44	0.46	0.48	0.36	0.43	0.43	0.30	0.39	0.37
PLH	0.41	0.44	0.45	0.53	0.58	0.57	0.34	0.46	0.44
YLD	0.42	0.44	0.46	0.49	0.55	0.55	0.43	0.48	0.49

**Table 9:** Mean heritability and accuracy of genomic prediction of traits across environments in cross validation.

	Heritability			Accuracy		
Model/Trait	Pedigree	Marker	Pedigree-Marker	Pedigree	Marker	Pedigree-Marker
DHE	0.50	0.48	0.48	0.36	0.42	0.42
DFL	0.70	0.70	0.69	0.40	0.47	0.47
DMA	0.27	0.30	0.29	0.26	0.31	0.31
PLH	0.87	0.89	0.88	0.40	0.46	0.47
YLD	0.64	0.57	0.58	0.36	0.42	0.42

Combining environments, the mean prediction accuracy of all traits was decreased in all models except for PLH for which accuracy was almost equal or higher than that in drought and heat stresses. The greatest reduction in accuracy occurred in the irrigated environment, which on average was 0.13% (0.09 to 0.22%) while the lowest reduction was observed under the heat stress by on average 0.03% (0 to 0.08%) (Table 9).

Combining environments also decreased the heritability of DHE and DMA compared to single environments in all models, while it increased the heritability of PLH. Furthermore, heritability of DFL was increased compared to heat and drought stresses but it decreased compared to irrigated environments. For YLD, heritability was lower for drought stress compared to irrigated and heat environments (Table 9).

## Discussion

Results of this study revealed that SYN parents are more diverse than cultivated BW wheat cultivars used in this study as shown in Fig 1. Also, based on Nei’s genetic diversity, SYN parents had higher genetic diversity than BW parents across all three genomes, specifically for D genome (*H*_*s*_ = 0.40) (Table 5). This was because 28 different *A*. *tauschii* accessions and 17 durums were used to develop the SYNs. The Nei’s genetic diversity indicated that SDL populations were more diverse than BW parents for A, B and D genomes in which D genome had the highest increased diversity (*H*_*s*_ = 0.19) (Table 5). Therefore, SYN lines are promising genetic resources to introduce novel genetic variation into the cultivated wheat gene pool. Similarly, Huang et al. [40] and Hoisington et al. [41] reported that SYN lines were used to improve quality, disease resistance, grain yield, and grain yield components of elite lines. One of the successful synthetic derived cultivars was Chuanmai-42 which increased grain yield by 0.45 to 0.75 t ha^-1^ in southwestern China compared to contemporary cultivars [2,14]. The SHW and SDLs are now widely used to develop modern wheat cultivars in China [2].

Equally important is the question of whether SYN lines can contribute to increased grain yield. The current study shows that the yield increases were predominantly in SDLs from BC1 derived lines (S5–S7 Tables). However, there were a few SDLs from biparental and TC crosses whose yield was higher than their corresponding BW parents. The potential of SDLs from BC1 derived lines to improve yield in both stress and normal conditions was reported in previous studies [12], [42,43] and [4]. However those studies did not have genotypes of the parents and derived lines.

Our results show that while SYN parents mostly have negative GEBVs for grain yield, they have less negative values under stress conditions and can increase grain yield of recurrent BW parents especially under drought and heat stress conditions (Fig 4A and S4 Table). Yield increases were more frequent under heat stress and the average yield GEBVs of the top 10% SDLs was 0.55 while for their recurrent BW parents it was 0.26 (Fig 5C1). Consequently, these results indicate that SYN lines are useful genetic resources for increasing grain yield in stress environments. Similar results were observed by Gororo et al.[44] who evaluated SDLs in drought and irrigated conditions and reported that SDLs exhibit higher yield potential over the recurrent parents in drought stress. Also, Reddy et al [45] evaluated common wheat lines and *T*. *tauschii* under drought stress and found that some *T*. *tauschii* lines represented were more tolerant than drought tolerant wheat lines. Furthermore, Ogbonnaya et al. [12] investigated the yield potential of SDLs (derived from BC1) in rainfed environments of Australia and reported that many of them out-yielded both recurrent parents and commercial varieties from 8 to 30% in different environments. They concluded that SDLs were able to improve yield in more diverse and stressed environments. For heat tolerance, Sharma et al. [46] evaluated 24 SYN lines under heat stress and identified three highly tolerant SYN lines. Using polymorphic inter-simple sequence repeat (ISSR) markers, they found that the genetic basis of heat tolerance in SYN lines is different and these new sources of genetic diversity could be used to improve heat tolerance of cultivated wheats. Furthermore, Cossani and Reynolds [47] by comparing six advanced synthetic derivative (ASD) lines with their BW and synthetic derivative (Syn-Der) parents under normal, heat-stress and extreme heat-stress envirnomemts reported that the ASD lines outperformed their best parent (Syn-Der) by on average 5, 15 and 13% for yield under normal, heat and extreme heat stress, respectively.

The higher yield of SDLs could be attributed to introgression of some positive alleles from the SYN lines that increase grain yield. For instance, Li et al. [15] used 705 polymorphic SSR markers and found four QTLs (*Barc1183*, *Barc241*, *Xcfe25*, *and Xcfd223*) from the SYN parent in Chuanmai-42 that had significant positive effects on grain yield. *Barc1183*, which is located on the long arm of chromosome 4D, increased grain yield by 7.00 to 11.30%. Similarly, Gororo et al. [44] investigated yield performance of SDLs derived from direct hybridization of wheat with *T*. *tauschii* and concluded that the increased yield in SDLs was caused by genes introduced from *T*. *tauschii*. Also, Liu et al [38] using introgression lines (ILs), crossed a SYN line, Am3, to common wheat, Laizhou953. Using 205 SSR markers they detected two QTLs (*Xgwm113* and *Xgwm159*) of Am3 on chromosomes 4B and 5B of the ILs that increased spikes per plant (0.65 to 1.18) and thousand kernel weight (6.10 to 6.30 gr), respectively. These findings support the introgression and retention of some positive yield QTLs from SYN lines in SDLs.

This study showed that the SYN lines contributed significantly more to increased grain yield of lower yielding BW parents in both stress and normal conditions. For example, SUNCO/2*PASTOR is one of the lower-yielding BW parents across all environments but all of its progenies that contributed to the top 10% of SDLs had higher GEBVs than the BW parents. Also of the high-yielding BW parents, MILAN/S87230//BAV92, produced 173 progenies among the top 10% SDLs, and 14% of them had higher GEBVs than the BW parent indicating that the SYN parents contributed positive alleles in crosses to all of the BWs.

In this study, SYN parents extended genetic diversity of the populations for three related traits, DHE, DFL and DMA, in the same direction across environments. As shown in Fig 5A2, 5B2 and 5C2, GEBVs of the top high-yielding SDLs for DMA are similar to the range of BW parent GEBVs. While the GEBVs of the SDLs are more diverse than those of the BW parents, the difference is small. This is because during segregating generations, populations were under selection for maturity approximating that of the BW parents. Since late maturing progenies were not included in the populations, these results did not represent the true diversity of the populations for these three traits. However, these results are likely to be more relevant to a wheat breeding program.

In this study there was a low correlation between GEBVs for yield and DMA, DFL and DHE, suggesting that the higher GEBVs of SDLs compared to their corresponding recurrent BW parents were not due to their phenology such as late or early maturity. This result differs from other studies. For example, Cooper et al. [13] reported that almost all high-yielding SDLs were earlier than their recurrent BW parents. In contrast to this study, they concluded that SYN lines contributed to yield because of their earlier maturity.

For PLH, diversity of populations was increased across environments (Fig 5A3, 5B3 and 5C3), but because of selection, diversity introduced from SYN lines was reduced. The GEBVs of top high-yielding SDLs for PLH were similar to those for BW parents in the heat stress (Fig 5B3), whereas most of them were taller than BW parents in irrigated and drought environments (Fig 5A3 and 5C3). Correlation coefficients of GEBVs of PLH and YLD were low across three environments (r = 0.04 to 0.29), suggesting that higher GEBVs of SDLs were not the result of increased plant height.

Our analyses showed that GFD values for SDLs were within the range of those for BW parents. However, this was due to selection of SDLs for maturity approximating that of the BW parents. So, these values did not show the true diversity of SYN lines for this trait. Also, the negative relationship between YLD and GFD indicated that there was no advantage of selecting genotypes for longer GFD. Increased YLD of SDLs was not associated with variation in GFD.

Results of this study indicated that SYN lines contributed to increased TKW of SDLs and increased the family mean from 0.67 to 24.39%. However, this contribution was not consistent for all SYN parents used in this study, such that family mean TKW of 11 SDLs were lower than the corresponding recurrent BW parents. Moreover, some SYN parents decreased TKW of SDLs in biparental populations while they increased TKW in the same BC populations. Our analyses for these specific populations indicated that, although 67% of SDLs had higher TKW than recurrent parents, the negative relationship and very low *R*^2^ values between TKW and YLD, indicated that phenotypic variation of YLD was not generally associated with TKW. Therefore, increased yield of SDLs was not a result of increased seed weight. In contrast to our finding, Cooper et al.[13] backcrossed ten elite primary synthetics to two Texas winter wheat cultivars, TAM111 and TAM112, and reported that all SYN lines contributed to high yielding SDLs through an increase in seed weight. Also, Röder et al [48], using ILs from crossing a synthetic line, W-7984, to a German winter wheat, ‘Prinz’, reported a QTL for grain weight, *QTgw*.*ipk-7D*, which was associated with microsatellite marker, *Xgwm1002-7D*. They reported that the ILs had 10% increased TKW compared to ‘Prinz’ and checks and 84.70% of the phenotypic variance could be explained by the segregation of *Xgwm1002-7D*.

### GEBV values of SYN lines and cultivated wheat

High-throughput genotyping technologies provide an opportunity to estimate breeding value of genotypes more accurately using a genomic relationship matrix [49]. These tools can improve the accuracy of parental selection in the breeding program. In this study, BW parents showed positive GEBVs for yield across all environments. Nevertheless, they reflected higher GEI in drought vs. irrigated, heat vs. irrigated, and drought vs. heat (Table 6). Some of the BW parents such as MILAN/S87230//BAV92 and BW line 3570 had high GEBV values in all environments (Fig 3A and S3 Table) and are good candidates to be used in breeding for diverse environments. On the other hand, almost all of the SYN lines had negative GEBVs across all environments for yield (Fig 4A and S4 Table). This was expected because SYN lines are exotic lines that have a durum variety and a wild diploid accession as parents and they have not been directly bred for yield. Only by evaluating populations of segregants from SYN crosses with BWs can we identify their positive and novel yield alleles for improving the yield of BW parents.

For PLH, most BW parents had negative GEBVs (Fig 3D and S3 Table) that can be attributed to dwarfing or semi-dwarfing genes in their genetic background. Generally, in irrigated environments plants with short to average height are favored to avoid lodging. Thus, parents with lower GEBVs for PLH are best suited for irrigated environments. Under stress conditions, taller plants are more tolerant as observed in this study (Fig 5B3 and 5C3). They can store more assimilates in their stems for remobilization during the grain filling stage. Thus, parents with high positive GEBVs would be better for production in stress environments. Although populations were under selection for PLH, all SYN lines had highly positive GEBV values for PLH (Fig 4D and S4 Table). This was because SYN lines are very tall genotypes and have many genes for PLH and selection did not remove all of them.

Our findings indicate that the majority of BW parents have positive GEBVs for DHE and DMA (Fig 3B and 3C), while nearly all SYN parents have negative GEBVs for DHE and decreased this trait (Fig 4B). For DMA, there are more SYN lines that have positive GEBVs in one or more environments (Fig 4C). We expected their positive GEBV values for these traits because SYN lines tend to be late maturing genotypes.

#### Genomic prediction

In traditional genetic evaluation, linear mixed models with the pedigree relationship matrix have been used for genomic prediction and selection in breeding programs [31] and [33]. New genotyping technologies provided dense genome-wide molecular markers that have been used to derive more accurate genomic relationships to increase selection accuracy in breeding programs [49–51] and [20]. Our results indicated that using marker data improved genomic prediction accuracy over the pedigree method. Improvement rates varied based on the different traits and environments and ranged from 2 to 12% (Table 8). The greatest improvement in prediction accuracy was mainly observed in heat stress (5 to 12%) and the lowest rate was observed in drought environments (2 to 5%) indicating that environments affect the relative prediction accuracy of pedigree—vs. marker—based prediction (Table 8). The higher prediction accuracy using the genomic relationship matrix is attributed to: 1) exploiting Mendelian sampling variation during gamete formation and 2) including relationship information from genotypes that the pedigree classified as unrelated genotypes 3) the **G** matrix provides better coverage of the genetic rearrangements that occur during SYN and SDL development that are not covered by the pedigree. [49,50] and [52]. A simulation study confirmed that using genomic relationship instead of pedigree relationship to estimate GEBVs increased selection accuracy [53]. Similar results were reported by Nejati-Javaremi [50] and VanRaden [30]. However, despite the potential mistakes in the pedigrees, genomic prediction accuracies from the pedigree model were reasonable and close to those of the marker model, in part because of the relatively small family sizes that limit the Mendelian segregation. This was because marker information was additionally used to identify incorrect pedigrees (removing outlier genotypes). In this study our results showed that, using the pedigree-marker method called the single–step blending approach by Gao et al.[31], that uses information from both genotyped and non-genotyped lines simultaneously to do genomic prediction did not improve genomic prediction accuracies (Tables 8 and 9).

Cross validation using combined environments decreased prediction accuracies of traits in all models (Table 9). However, the decreasing trend was not similar for all traits. The highest average decrease was observed for DHE and DMA (0.11%) while the lowest average reduction was for PLH (0.03%). These results were due to GEI interaction as shown in Tables 2,3 and 4 such that phenotypic correlations within treatments (irrigated, heat or drought) were overall greater than those among treatments. However, this was not consistent for all traits and for some of them among treatment correlations were greater than those for within treatments (e.g. PLH). These results confirmed that GEI affects the genomic prediction accuracy and traits with high GEI had lower prediction accuracy. Similarly, Zapata-Valenzuela et al. [52] argued that the accuracy of GEBVs using either **A** or **G** matrices would be lower in cases where there is strong GEI. This could lead to prediction models developed in one environment that lose their prediction power in other environments [52] and [54].

In this study estimated heritabilities using the pedigree model were consistently slightly higher than those using marker models (Table 7). This differences could be due to the differences in the amount of estimated genetic variances using **A** or **G** matrices in the model [37] as we observed in this study. Similarly, Loberg et al. [33] reported that the genetic variances estimated using the **A** matrix were greater than those estimated by the **G** matrix. Hence, estimated heritabilities using the **A** matrix were greater. They reported that gVarG, explained 10–60% of gVarA. Also, Powell et al [34] mentioned that incomplete linkage disequilibrium between the markers and the causal variants can reduce the genetic variance using the marker model. They concluded that the difference between the estimated gVarA and genetic variance explained by SNPs estimated using the **G** matrix was the missing heritability.

## Conclusion

These findings confirm that synthetic hexaploid wheat germplasm is a valuable genetic resource for improving grain yield and other traits. Synthetic hexaploid wheat lines have positive, novel alleles that can be easily introgressed into cultivated wheat to improve yield, especially in stress conditions. Therefore, SYN lines should be used in breeding programs to expand the genetic diversity for agronomic traits but selection against undesirable phenology is required to realize the benefit of the novel genetic variation.

## Supporting Information

S1 Table List of cultivated wheat and synthetic hexaploid lines used to develop the SDL populations. (PDF)

S2 Table TKW and GFD for BW parents, BP, BC and TC populations in IRRI.Y11.12. (PDF)

S3 Table GEBVs of cultivated wheat for measured traits in three contrasting environments. (PDF)

S4 Table GEBVs of SYN lines for measured traits in three contrasting environments. (PDF)

S5 Table GEBVs of BW parents and the top 10% of the SDLs within the population under heat stress. S5 Table compares GEBVs of BW parents (Gray row) with average GEBVs of its corresponding top 10% SDLs (White row) for grain yield (YLD) under heat stress.
(PDF)

S6 Table GEBVs of BW parents and the top 10% of the SDLs within the population under irrigated conditions. S6 Table compares GEBVs of BW parents (Gray row) with average GEBVs of its corresponding top 10% SDLs (White row) for grain yield (YLD) under irrigated condition.
(PDF)

S7 Table GEBVs of BW parents and the top 10% of the SDLs within the population under drought stress. S7 Table compares GEBVs of BW parents (Gray row) with average GEBVs of its corresponding top 10% SDLs (White row) for grain yield (YLD) under drought stress.
(PDF)
