# Supplementary material for: Breeding Value of Primary Synthetic Wheat Genotypes for Grain Yield
Source: PLoS One. 2016 Sep 22;11(9):e0162860. doi: 10.1371/journal.pone.0162860 (PMC5033409; doi:10.1371/journal.pone.0162860)
Supplement: S1 Table — (PDF) [file pone.0162860.s001.pdf]

**S1 Table. List of cultivated wheat and synthetic hexaploid lines used to develop the SDL populations.**	
GID	Bread Wheat parents
5653842	MILAN/S87230//BAV92
6763520	3570
6763547	CACUKE
6763623	KRL19
6763636	KIRITATI
6763790	PANDORA
6763911	KIRITATI/2*TRCH
6763917	SW89.5181/KAUZ
6764000	SUNCO/2*PASTOR
6764117	PBW502
6764191	MILAN/AMSEL
6764276	TAM200/TUI
6764815	MINO
6764954	MUU
6765077	HS420
6765130	KIRITATI//PRL/2*PASTOR
4747309	GONDO
4885783	GONDO//SHA5/WEAVER/3/PASTOR
4248	CNO79
	OCI

**S1 Table (continue). List of cultivated wheat and synthetic hexaploid lines used to develop the SDL populations.**		
GID	SYNP NO.	Synthetic parents
5989409	SYNP1	68.111/RGB-U//WARD RESEL/3/STIL/4/AE.SQUARROSA (1119)
5989410	SYNP2	68.111/RGB-U//WARD RESEL/3/STIL/4/AE.SQUARROSA (1164)
5989411	SYNP3	68.111/RGB-U//WARD RESEL/3/STIL/4/AE.SQUARROSA (1166)
5989414	SYNP4	68.111/RGB-U//WARD RESEL/3/STIL/4/AE.SQUARROSA (1219)
180150	SYNP5	68.111/RGB-U//WARD/3/AE.SQUARROSA (325)
5989418	SYNP6	68.111/RGB-U//WARD/3/FGO/4/RABI/5/AE.SQUARROSA (1110)
227752	SYNP7	6973/WARD.7463//74110/3/AE.SQUARROSA (438)
5989403	SYNP9	ALTAR 84/AE.SQUARROSA (895)
192513	SYNP11	CETA/AE.SQUAROOSA (263)
227787	SYNP12	CETA/AE.SQUARROSA (1055)
5989458	SYNP13	CETA/AE.SQUARROSA (1187)
5989460	SYNP14	CETA/AE.SQUARROSA (1219)
192496	SYNP15	CETA/AE.SQUARROSA (184)
5989431	SYNP16	CETA/AE.SQUARROSA (224)
5989440	SYNP17	CETA/AE.SQUARROSA (372)
5989446	SYNP18	CETA/AE.SQUARROSA (518)
5989450	SYNP19	CETA/AE.SQUARROSA (895)
5989390	SYNP20	CROC_1/AE.SQUARROSA (372)

5989393	SYNP21	CROC_1/AE.SQUARROSA (895)
174363	SYNP22	DOY1/AE.SQUARROSA (1027)
174317	SYNP23	DOY1/AE.SQUARROSA (334)
2447504	SYNP24	DOY1/AE.SQUARROSA (443)
180022	SYNP25	DVERD_2/AE.SQUARROSA (247)
172285	SYNP26	GAN/AE.SQUARROSA (446)
2447515	SYNP27	GAN/AE.SQUARROSA (536)
3562369	SYNP28	GARZA/BOY//AE.SQUARROSA (228)
5989495	SYNP31	LOCAL RED/AE.SQUARROSA (518)
5989487	SYNP34	RASCON_37/AE.SQUARROSA (205)
4061131	SYNP35	SHAG_22/AE.SQUARROSA (1084)
227726	SYNP36	SHAG_22/AE.SQUARROSA (239)
5989505	SYNP39	SOMAT_4/INTER_8//AE.SQUARROSA (1206)
172922	SYNP43	YUK/AE.SQUARROSA (864)
4254403	SYNP44	MAYOOR//TK SN1081/AE.SQUARROSA (222)/3/OCI
