# Supplementary material for: Breeding Value of Primary Synthetic Wheat Genotypes for Grain Yield
Source: PLoS One. 2016 Sep 22;11(9):e0162860. doi: 10.1371/journal.pone.0162860 (PMC5033409; doi:10.1371/journal.pone.0162860)
Supplement: S2 Table — (PDF) [file pone.0162860.s002.pdf]

S2 Table. TKW and GFD for BW parents, BP, BC and TC populations in IRRI.Y11.12.

Populations	Cross [1]	BW[2] parents	SYN[3] parents	Mean GFD[4] (days)	Range GFD (days)	BWP GFD (days)	Increase/decrease GFD (%)	Mean TKW[5] (gr)	Range TKW (gr)	BWP TKW (gr)	Increase/decrease TKW (%)
68	BP	CACUKE	SYNP16	56	54-59	57	-1.8	52	41-61	54	-3.70
60	BP	CACUKE	SYNP5	58.5	57-60	57	2.6	57.8	51-63	54	7.04
78	BP	KIRITATI	SYNP24	55	55-55	52	5.8	49	49-49	50	-2.00
71	BP	KRL19	SYNP18	54.5	54-55	56	-2.7	44	44-44	41	7.32
86	BP	KRL19	SYNP36	58	58-58	56	3.6	51	51-51	41	24.39
70	BP	MILAN/S87230//BAV92	SYNP17	53.5	53-54	52	2.9	46.5	46-47	43	8.14
75	BP	MILAN/S87230//BAV92	SYNP20	52.9	49-56	52	1.7	52	44-59	43	20.93
77	BP	MILAN/S87230//BAV92	SYNP21	54.8	52-57	52	5.4	51.5	46-57	43	19.77
83	BP	MILAN/S87230//BAV92	SYNP27	51	49-54	52	-1.9	48	44-51	43	11.63
88	BP	MILAN/S87230//BAV92	SYNP39	53.3	50-57	52	2.5	50.8	43-60	43	18.14
58	BP	MILAN/S87230//BAV92	SYNP4	56	54-57	52	7.7	51	49-54	43	18.60
84	BP	MUU	SYNP34	55.5	50-60	51	8.8	53	42-62	47	12.77
67	BP	PANDORA	SYNP14	56.2	55-60	55	2.2	50.6	45-60	45	12.44
72	BP	PANDORA	SYNP18	55.8	52-59	55	1.5	46.6	46-48	45	3.56
73	BP	PANDORA	SYNP19	55.6	53-58	55	1.1	50.8	48-55	45	12.89
53	BP	PANDORA	SYNP1	53.3	50-56	55	-3.1	45.3	44-46	45	0.67
76	BP	PANDORA	SYNP21	57.3	56-61	55	4.2	55.5	50-64	45	23.33
54	BP	PANDORA	SYNP2	57	54-60	55	3.6	43.5	43-44	45	-3.33
89	BP	PANDORA	SYNP39	54.1	50-56	55	-1.6	48	45-51	45	6.67
56	BP	PANDORA	SYNP3	58	58-58	55	5.5	51	51-51	45	13.33
80	BP	PBW502	SYNP25	61	61-61	52	17.3	58	58-58	51	13.73
90	BP	PBW502	SYNP43	58	58-58	52	11.5	52	52-52	51	1.96
61	BP	PBW502	SYNP5	58	56-60	52	11.5	49	49-49	51	-3.92
82	BP	SUNCO/2*PASTOR	SYNP27	53.4	51-56	53	0.8	46	42-50	41	12.20
62	BP	SUNCO/2*PASTOR	SYNP5	56.7	56-57	53	7.0	47	43-49	41	14.63
63	BP	SW89.5181/KAUZ	SYNP6	50.3	49-52	49	2.7	46	43-48	43	6.98

[1]: Cross; BP: Bi-parental, BC: Back-cross, TC: Three-way cross, [2]: BW: Bread wheat, [3]: SYN; Synthetic, [4]: GFD; Grain filling duration, [5]: TKW; Thousand kernel weight.

S2 Table. (Continue) TKW and GFD for BW parents, BP, BC and TC populations in IRRI.Y11.12.											
Populations	Cross	BW parents	SYN parents	Mean GFD (days)	Range GFD (days)	BWP GFD (days)	Increase/decrease GFD (%)	Mean TKW (gr)	Range TKW (gr)	BWP TKW (gr)	Increase/decrease TKW (%)
22	BC	CACUKE	SYNP16	57	55-59	57	0.0	55.8	50-61	54	3.33
35	BC	CACUKE	SYNP25	56.9	55-58	57	-0.2	56.6	51-62	54	4.81
48	BC	CACUKE	SYNP43	57.7	55-60	57	1.2	56.9	51-64	54	5.37
9	BC	CACUKE	SYNP5	57.5	56-59	57	0.9	57	51-65	54	5.56
16	BC	KIRITATI	SYNP11	51.7	49-53	52	-0.6	47.3	44-49	50	-5.40
34	BC	KIRITATI	SYNP24	53.5	51-55	52	2.9	52.2	47-57	50	4.40
37	BC	KIRITATI	SYNP26	52.7	50-55	52	1.3	48.5	45-53	50	-3.00
10	BC	KIRITATI	SYNP5	55.2	53-58	52	6.2	47.3	41-56	50	-5.40
94	TC	KIRITATI//PRL/2*PASTOR	SYNP44	52.1	51-54	52	0.2	44.5	40-53	54	-17.59
92	TC	KRL19	SYNP15	55	51-59	56	-1.8	43.8	42-46	41	6.83
25	BC	KRL19	SYNP18	57.1	55-59	56	2.0	45.6	42-51	41	11.22
45	BC	KRL19	SYNP36	56.9	53-60	56	1.6	45.9	43-54	41	11.95
91	TC	MILAN/AMSEL	SYNP7	53.9	49-57	52	3.7	48.6	43-55	44	10.45
24	BC	MILAN/S87230//BAV92	SYNP17	51.5	48-54	52	-1.0	46.7	42-56	43	8.60
29	BC	MILAN/S87230//BAV92	SYNP20	52.6	50-56	52	1.2	47.2	41-57	43	9.77
31	BC	MILAN/S87230//BAV92	SYNP21	53.4	50-56	52	2.7	48.8	41-57	43	13.49
33	BC	MILAN/S87230//BAV92	SYNP23	53.3	50-56	52	2.5	46.7	42-53	43	8.60
40	BC	MILAN/S87230//BAV92	SYNP27	53	49-57	52	1.9	47.2	43-55	43	9.77
47	BC	MILAN/S87230//BAV92	SYNP39	52.9	48-56	52	1.7	46.3	40-52	43	7.67
7	BC	MILAN/S87230//BAV92	SYNP4	53.8	51-57	52	3.5	47.5	42-57	43	10.47
96	TC	MINO	SYNP36	55.8	54-59	54	3.3	46.2	41-57	41	12.68
43	BC	MUU	SYNP34	52.4	51-54	51	2.7	46	41-49	47	-2.13
17	BC	PANDORA	SYNP11	55.2	54-57	55	0.4	44.8	41-51	45	-0.44
21	BC	PANDORA	SYNP14	56.1	52-60	55	2.0	49.1	42-53	45	9.11
26	BC	PANDORA	SYNP18	54.3	53-56	55	-1.3	46.6	42-51	45	3.56
27	BC	PANDORA	SYNP19	56.2	55-57	55	2.2	48.2	42-54	45	7.11
2	BC	PANDORA	SYNP1	54.3	51-57	55	-1.3	46.9	42-52	45	4.22
30	BC	PANDORA	SYNP21	55.6	54-58	55	1.1	51.7	47-59	45	14.89

S2 Table. (Continue) TKW and GFD for BW parents, BP, BC and TC populations in IRRI.Y11.12.											
**Populations**	**Cross**	**BW parents**	**SYN parents**	**Mean GFD (days)**	**Range GFD (days)**	**BWP GFD (days)**	**Increase/decrease GFD (%)**	**Mean TKW (gr)**	**Range TKW (gr)**	**BWP TKW (gr)**	**Increase/decrease TKW (%)**
32	BC	PANDORA	SYNP23	55	53-57	55	0.0	47.7	42-51	45	6.00
38	BC	PANDORA	SYNP26	55.9	52-58	55	1.6	48	41-53	45	6.67
3	BC	PANDORA	SYNP2	56	56-56	55	1.8	44.7	42-49	45	-0.67
42	BC	PANDORA	SYNP31	55	53-56	55	0.0	50.3	44-57	45	11.78
46	BC	PANDORA	SYNP39	55.2	53-58	55	0.4	45.8	40-53	45	1.78
5	BC	PANDORA	SYNP3	56.2	54-58	55	2.2	51.4	47-59	45	14.22
36	BC	PBW502	SYNP25	59.3	58-61	52	14.0	54.5	49-60	51	6.86
49	BC	PBW502	SYNP43	59.5	57-62	52	14.4	55.8	51-61	51	9.41
11	BC	PBW502	SYNP5	58.9	57-61	52	13.3	51.7	45-65	51	1.37
39	BC	SUNCO/2*PASTOR	SYNP27	53.7	51-58	53	1.3	47.2	43-54	41	15.12
50	BC	SUNCO/2*PASTOR	SYNP43	55.3	52-59	53	4.3	47.6	43-57	41	16.10
12	BC	SUNCO/2*PASTOR	SYNP5	56.2	52-61	53	6.0	45.9	41-50	41	11.95
44	BC	SW89.5181/KAUZ	SYNP35	52.3	49-57	49	6.7	46.5	40-56	43	8.14
14	BC	SW89.5181/KAUZ	SYNP6	51.4	48-57	49	4.9	44.6	41-52	43	3.72
