# Supplementary material for: Breeding Value of Primary Synthetic Wheat Genotypes for Grain Yield
Source: PLoS One. 2016 Sep 22;11(9):e0162860. doi: 10.1371/journal.pone.0162860 (PMC5033409; doi:10.1371/journal.pone.0162860)
Supplement: S3 Table — (PDF) [file pone.0162860.s003.pdf]

S3 Table. GEBVs of cultivated wheat for measured traits in three contrasting environments.

Trait	YLD			PLH			DHE			DFL			DMA		
Environments BWP	IRRI.	DRO.	HEAT	IRRI.	DRO.	HEAT	IRRI.	DRO.	HEAT	IRRI.	DRO.	HEAT	IRRI.	DRO.	HEAT
CNO79	0.482	0.116	0.100	-11.843	-4.774	-2.542	3.611	2.553	0.603	3.035	2.364	1.170	1.953	1.579	0.464
GONDO	0.162	-0.147	0.210	-3.638	-2.789	0.089	4.521	3.279	0.623	6.201	2.921	2.782	3.572	3.296	0.447
GONDO//SHA5/WEAVER/3/PASTOR	0.394	0.097	0.030	-7.832	-4.536	-3.451	0.908	1.733	0.197	1.989	1.563	0.130	1.462	0.543	-0.220
MILAN/S87230//BAV92	1.400	0.435	0.647	-1.926	-2.664	-0.163	3.529	1.008	0.363	2.735	0.851	1.622	0.284	0.316	0.161
BW Line 3570	1.344	0.416	0.643	-1.720	-2.542	-0.139	3.663	1.084	0.502	2.849	0.828	1.725	0.370	0.369	0.296
CACUKE	0.427	0.132	0.147	1.276	3.656	2.295	-4.237	-0.941	-2.050	-2.984	-0.261	-2.903	-2.160	-0.182	-1.263
KRL19	0.458	0.212	0.251	-10.248	-4.009	-1.304	-3.341	-0.624	-1.504	-2.901	-0.480	-1.980	-2.091	-1.258	-1.335
KIRITATI	0.824	0.226	0.422	-1.556	-0.152	1.740	5.033	1.967	1.469	3.302	0.842	2.324	1.570	0.718	0.913
PANDORA	0.741	0.078	-0.011	-8.458	-4.549	-4.306	2.716	3.731	1.720	3.024	3.171	1.585	2.523	2.376	0.786
KIRITATI/2*TRCH	-0.067	0.283	0.282	-12.240	-4.633	-5.656	-7.833	-3.893	0.234	-5.538	-2.449	-1.693	-3.046	-4.821	-0.377
SW89.5181/KAUZ	0.906	-0.128	0.003	-4.146	-5.945	-4.030	9.883	5.608	5.047	7.713	4.233	5.046	4.279	2.906	2.951
SUNCO/2*PASTOR	-0.160	0.117	0.110	0.548	-2.816	-1.917	3.014	1.122	2.357	1.712	1.175	1.101	1.242	-0.166	2.444
PBW502	0.633	0.146	0.650	-0.536	-2.916	5.560	4.328	2.078	0.476	3.775	2.154	2.656	1.624	1.320	0.356
MILAN/AMSEL	0.165	-0.111	-0.033	-3.063	-3.320	-1.316	6.567	3.358	0.109	5.312	3.162	0.790	3.155	2.453	-0.111
TAM200/TUI	0.466	0.007	0.253	-5.665	-3.619	-1.351	-0.400	0.858	1.567	0.453	0.996	1.901	0.213	-0.361	0.684
MINO	0.475	0.263	0.242	-2.680	1.615	2.446	1.243	3.810	-2.333	0.948	2.427	-1.272	0.366	1.131	-1.438
MUU	-0.021	0.026	-0.326	1.560	-2.548	-1.257	5.019	1.901	2.385	2.780	1.119	2.974	1.108	0.598	1.077
HS420	0.848	0.098	0.244	-3.405	-4.523	-2.199	0.246	0.702	-0.506	2.680	1.772	-0.802	2.496	3.019	-0.625
KIRITATI//PRL/2*PASTOR	0.764	0.205	0.342	-2.483	-0.469	1.247	4.457	1.861	1.475	2.905	0.793	2.357	1.681	0.809	0.928
