# Supplementary material for: Breeding Value of Primary Synthetic Wheat Genotypes for Grain Yield
Source: PLoS One. 2016 Sep 22;11(9):e0162860. doi: 10.1371/journal.pone.0162860 (PMC5033409; doi:10.1371/journal.pone.0162860)
Supplement: S4 Table — (PDF) [file pone.0162860.s004.pdf]

S4 Table. GEBVs of SYN lines for measured traits in three contrasting environments.

Trait	YLD			PLH			DHE			DFL			DMA		
Environments<br>SYN parent NO.	IRRI.	DRO.	HEAT	IRRI.	DRO.	HEAT	IRRI.	DRO.	HEAT	IRRI.	DRO.	HEAT	IRRI.	DRO.	HEAT
SYNP1	-2.46	-0.55	-1.74	19.84	9.69	3.42	1.30	-1.80	0.62	-0.41	-2.23	-1.98	-0.13	-1.28	0.51
SYNP2	-2.28	-0.64	-1.06	15.65	8.28	4.83	0.07	-3.68	0.25	-1.86	-3.39	-1.30	-0.81	-1.52	0.54
SYNP3	-2.07	-0.35	-0.85	20.51	11.91	5.49	-1.20	-3.14	-0.34	-0.39	-3.32	-2.41	0.65	-0.88	0.07
SYNP4	-1.69	-0.34	-0.58	17.92	10.20	3.95	-2.13	-2.91	-0.26	-1.03	-2.96	-2.51	1.51	-0.13	0.32
SYNP5	-0.25	-0.15	-0.39	1.76	4.31	-0.55	-8.04	-5.06	-2.99	-5.49	-4.07	-3.83	-0.85	-1.68	-1.67
SYNP6	-2.46	-0.52	-0.81	16.20	8.58	3.30	0.22	-2.68	1.21	-1.85	-2.42	0.50	-3.50	-2.95	1.33
SYNP7	-0.73	-0.24	-0.09	1.20	2.94	0.54	-4.92	-3.19	-2.68	-1.46	-1.27	-1.71	0.95	1.08	-1.52
SYNP9	-1.60	-0.42	-0.39	12.40	5.30	3.49	-1.01	-1.07	-0.43	0.57	-0.81	-2.41	2.62	1.21	0.18
SYNP11	-1.45	-0.66	-0.81	5.66	2.54	2.40	-0.31	-1.62	-0.24	-1.79	-1.51	-1.32	-1.18	-0.57	-0.01
SYNP12	-1.21	-0.31	0.26	7.04	3.06	1.84	-6.08	-6.08	-4.19	-5.00	-2.67	-1.28	-1.71	-1.15	-1.94
SYNP13	-0.94	-0.32	-0.10	12.05	6.73	3.32	-5.23	-3.97	-2.44	-2.30	-3.35	-3.41	0.64	-1.23	-1.42
SYNP14	-1.55	-0.34	-0.02	20.46	10.44	5.63	-4.16	-3.41	-3.07	-1.76	-3.37	-2.86	-0.34	-0.25	-0.88
SYNP15	-0.49	-0.26	-0.05	13.25	4.69	3.01	-1.86	-0.70	-3.07	-0.24	-0.06	-1.35	0.43	1.35	-1.06
SYNP16	-2.05	-0.52	-0.71	10.09	4.41	5.31	-1.16	-1.44	-2.18	-1.59	-2.24	-2.36	-0.22	-0.90	-0.58
SYNP17	-1.88	-0.76	-1.01	20.10	7.80	4.60	-0.67	-1.61	-2.03	-2.19	-2.19	-1.48	-1.12	-0.09	0.37
SYNP18	-2.38	-0.67	-0.69	17.85	7.45	5.42	-0.42	-3.93	-1.10	-0.92	-3.16	-1.53	-1.98	-1.77	-0.21
SYNP19	-1.54	-0.44	-0.28	16.28	8.18	5.84	-0.88	-2.41	-1.77	-0.82	-2.36	-2.51	0.61	0.04	-0.24
SYNP20	-1.89	-1.02	-1.15	8.20	1.52	2.84	0.54	-0.22	-0.04	2.37	0.14	-0.66	2.24	1.87	1.12
SYNP21	-1.62	-0.60	-0.41	5.82	3.69	4.53	-1.44	-1.02	-0.57	2.11	-0.56	-2.21	3.04	1.45	0.30
SYNP22	-1.00	-0.33	-0.16	9.98	4.11	3.00	-4.30	-3.62	-2.67	-3.13	-2.99	-2.56	0.00	-2.01	-1.68
SYNP23	-1.39	-0.44	-0.54	3.43	1.68	2.61	-1.54	-1.84	-1.61	-0.51	-1.48	-2.04	0.70	-0.27	-0.76
SYNP24	-1.19	-0.38	-0.25	12.80	6.77	3.74	-4.46	-3.92	-2.13	-2.93	-3.41	-2.85	-0.61	-1.86	-1.25
SYNP25	-1.80	-0.36	-0.22	13.12	10.70	4.48	-6.90	-6.48	-4.31	-2.97	-4.64	-3.57	-1.81	-3.46	-2.50
SYNP26	-0.44	-0.10	0.09	6.44	4.30	1.37	-4.92	-4.62	-2.47	-4.24	-2.75	-2.82	0.67	-1.17	-1.19
SYNP27	-1.24	-0.14	0.14	0.58	2.82	0.02	-5.42	-5.34	-1.67	-3.21	-2.21	0.28	-1.62	-2.24	-0.57
SYNP28	-2.69	-0.63	-0.72	7.54	6.55	1.67	-3.91	-5.75	-1.14	-1.69	-3.41	-1.96	-1.15	-2.54	-0.55
SYNP31	-2.43	-0.63	-0.85	15.18	4.76	3.91	-2.13	-2.51	0.56	-4.07	-3.49	-1.39	-1.52	-0.61	0.12
SYNP34	-2.02	-0.51	-0.99	6.43	2.37	5.11	-1.14	-0.67	0.12	-0.29	-0.68	-1.20	1.16	1.17	0.72
SYNP35	-2.20	-0.66	-0.57	9.26	5.64	3.76	-5.26	-6.61	-1.65	-2.12	-3.56	-2.60	-2.12	-2.43	-0.68
SYNP36	-1.21	-0.32	0.24	7.94	3.35	1.47	-5.92	-5.99	-4.07	-4.60	-2.66	-1.30	-1.29	-1.16	-1.82
SYNP39	-2.09	-0.37	-0.56	7.36	4.97	4.04	-0.46	-1.34	-0.16	0.35	-0.62	-0.92	-0.90	-0.47	0.03
SYNP43	-1.06	-0.28	-0.17	9.96	4.72	4.12	-4.97	-3.60	-0.25	-5.26	-3.54	-1.12	0.12	-1.72	0.09
SYNP44	-0.29	-0.10	-0.16	-6.53	-3.21	-0.70	1.55	1.48	0.06	1.99	1.72	0.14	1.79	1.26	-0.14
