# Supplementary material for: Breeding Value of Primary Synthetic Wheat Genotypes for Grain Yield
Source: PLoS One. 2016 Sep 22;11(9):e0162860. doi: 10.1371/journal.pone.0162860 (PMC5033409; doi:10.1371/journal.pone.0162860)
Supplement: S5 Table — compares GEBVs of BW parents (Gray row) with average GEBVs of its corresponding top 10% SDLs (White row) for grain yield (YLD) under heat stress. (PDF) [file pone.0162860.s005.pdf]

S5 Table. GEBVs of BW parents and the top 10% of the SDLs within the population under heat stress.				
BW Parents	SYN Parents	Cross	Ave. Yield GEBV	% increase/decrease GEBV
**3570**			**0.64**	
3570	SYNP14	**BC**	**0.55**	**-14**
**CACUKE**			**0.15**	
CACUKE	SYNP5	BC	0.46	215
CACUKE	SYNP16	BC	0.50	243
CACUKE	SYNP43	BC	0.62	322
**GONDO// SHA5/WEAVER/3/PASTOR**			**0.03**	
GONDO// SHA5/WEAVER/3/PASTOR	SYNP7	BP	0.45	42
**HS420**			**0.24**	
HS420	SYNP13	BC	0.59	142
**KIRITATI**			**0.42**	
KIRITATI	SYNP5	BC	0.43	2
**KRL19**			**0.25**	
KRL19	SYNP18	BC	0.49	95
KRL19	SYNP36	BC	0.42	67
**MILAN/S87230//BAV92**			**0.65**	
MILAN/S87230//BAV92	SYNP4	BC	0.59	-9
MILAN/S87230//BAV92	SYNP4	BP	0.45	-30
MILAN/S87230//BAV92	SYNP17	BC	0.49	-24
MILAN/S87230//BAV92	SYNP17	BP	0.57	-12
MILAN/S87230//BAV92	SYNP20	BC	0.53	-18
MILAN/S87230//BAV92	SYNP20	BP	0.41	-37
MILAN/S87230//BAV92	SYNP21	BP	0.61	-6
MILAN/S87230//BAV92	SYNP21	BC	0.62	-4
MILAN/S87230//BAV92	SYNP23	BC	0.58	-10
MILAN/S87230//BAV92	SYNP27	BC	0.58	-10
MILAN/S87230//BAV92	SYNP39	BC	0.51	-21
MILAN/S87230//BAV92	SYNP39	BP	0.49	-24
**MINO**			**0.24**	
MINO	SYNP36/4/GONDO// SHA5/WEAVER/3/PASTOR	TC	0.50	108
**MUU**			**-0.33**	
MUU	SYNP34	BP	0.55	69
**PANDORA**			**-0.01**	
PANDORA	SYNP1	BC	0.64	63
PANDORA	SYNP14	BP	0.59	58
PANDORA	SYNP19	BP	0.43	42
PANDORA	SYNP19	BC	0.46	45
PANDORA	SYNP21	BP	0.53	52
PANDORA	SYNP26	BC	0.49	48
**PBW502**			**0.65**	
PBW502	SYNP22//KIRITATI	TC	0.48	-26
PBW502	SYNP5	BC	0.42	-35
PBW502	SYNP25	BC	0.52	-20
**SUNCO/2*PASTOR**			**0.11**	
SUNCO/2*PASTOR	SYNP27	BC	0.58	427
SUNCO/2*PASTOR	SYNP5	BC	0.47	327
SUNCO/2*PASTOR	SYNP43	BC	0.49	345
**TAM200/TUI**			**0.25**	
TAM200/TUI	SYNP2	BC	0.48	92
TAM200/TUI	SYNP3	BP	0.41	64
TAM200/TUI	SYNP4	BC	0.42	68
