# Supplementary material for: Breeding Value of Primary Synthetic Wheat Genotypes for Grain Yield
Source: PLoS One. 2016 Sep 22;11(9):e0162860. doi: 10.1371/journal.pone.0162860 (PMC5033409; doi:10.1371/journal.pone.0162860)
Supplement: S6 Table — compares GEBVs of BW parents (Gray row) with average GEBVs of its corresponding top 10% SDLs (White row) for grain yield (YLD) under irrigated condition. (PDF) [file pone.0162860.s006.pdf]

S6 Table. GEBVs of BW parents and their SDLs in the top 10% of the population under irrigated conditions.

BW Parents	SYN Parents	Cross	Ave. Yield GEBVs	% increase/decrease GEBV
**3570**			**1.34**	
3570	SYNP14	BC	0.98	-27
**CACUKE**			**0.43**	
CACUKE	SYNP5	BC	0.73	72
**HS420**			**0.85**	
HS420	SYNP13	BC	0.78	-8
**KIRITATI**			**0.82**	
KIRITATI	SYNP5	BC	0.78	-6
**MILAN/S87230//BAV92**			**1.40**	
MILAN/S87230//BAV92	SYNP4	BC	0.92	-34
MILAN/S87230//BAV92	SYNP4	BP	0.78	-44
MILAN/S87230//BAV92	SYNP20	BC	0.94	-33
MILAN/S87230//BAV92	SYNP20	BP	0.89	-37
MILAN/S87230//BAV92	SYNP21	BC	0.90	-36
MILAN/S87230//BAV92	SYNP21	BP	0.83	-41
MILAN/S87230//BAV92	SYNP21	BP	0.86	-38
MILAN/S87230//BAV92	SYNP23	BC	1.02	-27
MILAN/S87230//BAV92	SYNP27	BC	1.05	-25
MILAN/S87230//BAV92	SYNP27	BP	0.78	-45
MILAN/S87230//BAV92	SYNP27	BP	0.72	-49
MILAN/S87230//BAV92	SYNP39	BC	0.86	-38
**MINO**			**0.48**	
MINO	SYNP36/4/GONDO//SHA5/WEAVER/3/PASTOR	TC	0.84	77
**MUU**			**-0.02**	
MUU	SYNP34	BP	0.76	78
**PANDORA**			**0.74**	
PANDORA	SYNP3	BC	1.00	35
PANDORA	SYNP11	BC	0.72	-2
PANDORA	SYNP14	BC	0.80	8
PANDORA	SYNP18	BP	0.83	12
PANDORA	SYNP19	BC	0.96	29
PANDORA	SYNP23	BC	0.71	-4
PANDORA	SYNP26	BC	0.82	11
PANDORA	SYNP39	BC	0.69	-7
**SUNCO/2*PASTOR**			**-0.16**	
SUNCO/2*PASTOR	SYNP5	BC	0.95	111
SUNCO/2*PASTOR	SYNP5	BP	0.71	87
SUNCO/2*PASTOR	SYNP27	BC	0.80	96
SUNCO/2*PASTOR	SYNP43	BC	0.90	106
**SW89.5181/KAUZ**			**0.91**	
SW89.5181/KAUZ	SYNP6	BC	1.09	20
SW89.5181/KAUZ	SYNP35	BC	0.76	-17
**TAM200/TUI**			**0.47**	
TAM200/TUI	SYNP2	BC	0.78	67
TAM200/TUI	SYNP3	BC	0.80	71
