# Supplementary material for: Breeding Value of Primary Synthetic Wheat Genotypes for Grain Yield
Source: PLoS One. 2016 Sep 22;11(9):e0162860. doi: 10.1371/journal.pone.0162860 (PMC5033409; doi:10.1371/journal.pone.0162860)
Supplement: S7 Table — compares GEBVs of BW parents (Gray row) with average GEBVs of its corresponding top 10% SDLs (White row) for grain yield (YLD) under drought stress. (PDF) [file pone.0162860.s007.pdf]

**S7 Table. GEBVs of BW parents and their SDLs in the top 10% of the population under drought stress.**				
BW Parents	SYN Parents	Cross	Ave. Yield GEBVs	% increase/decrease GEBVs
**CACUKE**			**0.13**	
CACUKE	SYNP43	BC	0.27	104
**CNO79**			**0.12**	
CNO79	SYNP44	TC	0.32	177
**KIRITATI**			**0.23**	
KIRITATI	SYNP5	BC	0.31	36
**KIRITATI/2*TRCH**			**0.28**	
KIRITATI/2*TRCH	SYNP1	BC	0.32	12
**KRL19**			**0.21**	
KRL19	SYNP18	BC	0.33	53
**MILAN/AMSEL**			**-0.11**	
MILAN/AMSEL	SYNP7/4/GONDO//SHA5/WEAVER/3/PASTOR	TC	0.34	411
**MILAN/S87230//BAV92**			**0.44**	
MILAN/S87230//BAV92	SYNP4	BC	0.35	-20
MILAN/S87230//BAV92	SYNP4	BP	0.32	-27
MILAN/S87230//BAV92	SYNP17	BP	0.41	-5
MILAN/S87230//BAV92	SYNP21	BC	0.34	-23
MILAN/S87230//BAV92	SYNP21	BP	0.29	-33
MILAN/S87230//BAV92	SYNP23	BC	0.34	-21
MILAN/S87230//BAV92	SYNP27	BC	0.38	-12
MILAN/S87230//BAV92	SYNP27	BP	0.34	-22
MILAN/S87230//BAV92	SYNP39	BP	0.44	0
MILAN/S87230//BAV92	SYNP39	BC	0.34	-21
MILAN/S87230//BAV92	SYNP39	BP	0.39	-10
**MINO**			**0.26**	
MINO	SYNP36/4/GONDO//SHA5/WEAVER/3/PASTOR	TC	0.32	22
**PANDORA**			**0.08**	
PANDORA	SYNP1	BC	0.30	282
PANDORA	SYNP3	BC	0.27	252
PANDORA	SYNP14	BP	0.41	422
PANDORA	SYNP18	BP	0.28	260
PANDORA	SYNP19	BC	0.27	252
PANDORA	SYNP31	BC	0.26	239
PANDORA	SYNP39	BP	0.28	259
**PBW502**			**0.15**	
PBW502	SYNP5	BC	0.34	134
PBW502	SYNP25	BC	0.27	82
PBW502	SYNP43	BC	0.31	110
**SUNCO/2*PASTOR**			**0.12**	
SUNCO/2*PASTOR	SYNP5	BC	0.35	196
SUNCO/2*PASTOR	SYNP27	BC	0.37	218
SUNCO/2*PASTOR	SYNP43	BC	0.33	182
**SW89.5181/KAUZ**			**-0.13**	
SW89.5181/KAUZ	SYNP35	BC	0.34	363
